# Enhancing adoptive cancer immunotherapy with Vγ2Vδ2 T cells through pulse zoledronate stimulation

**DOI:** 10.1186/s40425-017-0209-6

**Published:** 2017-02-21

**Authors:** Mohanad H. Nada, Hong Wang, Grefachew Workalemahu, Yoshimasa Tanaka, Craig T. Morita

**Affiliations:** 1 Division of Immunology, Department of Internal Medicine, University of Iowa Carver College of Medicine, Iowa City, IA 52242 USA; 2 Department of Veterans Affairs, Iowa City Health Care System, Iowa City, IA 52246 USA; 3 Interdisciplinary Graduate Program in Immunology, University of Iowa Carver College of Medicine, Iowa City, IA 52242 USA; 4 Department of Pathology, College of Medicine, Tikrit University, Tikrit, Iraq; 5 Center for Bioinformatics and Molecular Medicine, Graduate School of Biomedical Sciences, Nagasaki University, 1-12-4 Sakamoto, Nagasaki, 852-8523 Japan

**Keywords:** γδ T cells, Vγ2Vδ2 T cells, Human, Bisphosphonate, Zoledronate, Adoptive cancer immunotherapy, Prostate cancer, IL-2, IL-15, Memory T cell subsets

## Abstract

**Background:**

Human γδ T cells expressing Vγ2Vδ2 T cell receptors monitor foreign- and self-prenyl pyrophosphate metabolites in isoprenoid biosynthesis to mediate immunity to microbes and tumors. Adoptive immunotherapy with Vγ2Vδ2 T cells has been used to treat cancer patients with partial and complete remissions. Most clinical trials and preclinical studies have used continuous zoledronate exposure to expand Vγ2Vδ2 cells where zoledronate is slowly diluted over the course of the culture. Zoledronate inhibits farnesyl diphosphate synthase (FDPS) in monocytes causing isopentenyl pyrophosphate to accumulate that then stimulates Vγ2Vδ2 cells. Because zoledronate inhibition of FDPS is also toxic for T cells, we hypothesized that a short period of exposure would reduce T cell toxicity but still be sufficient for monocytes uptake. Additionally, IL-15 increases the anti-tumor activity of murine αβ T cells in mice but its effect on the in vivo anti-tumor activity of human Vγ2Vδ2 cells has not been assessed.

**Methods:**

Human Vγ2Vδ2 T cells were expanded by pulse or continuous zoledronate stimulation with IL-2 or IL-15. Expanded Vγ2Vδ2 cells were tested for their expression of effector molecules and killing of tumor cells as well as their in vivo control of human prostate cancer tumors in immunodeficient NSG mice.

**Results:**

Pulse zoledronate stimulation with either IL-2 or IL-15 resulted in more uniform expansion of Vγ2Vδ2 cells with higher purity and cell numbers as compared with continuous exposure. The Vγ2Vδ2 cells had higher levels of CD107a and perforin and increased tumor cytotoxicity. Adoptive immunotherapy with Vγ2Vδ2 cells derived by pulse stimulation controlled human PC-3 prostate cancer tumors in NSG mice significantly better than those derived by continuous stimulation, halting tumor growth. Although pulse zoledronate stimulation with IL-15 preserved early memory subsets, adoptive immunotherapy with IL-15-derived Vγ2Vδ2 cells equally inhibited PC-3 tumor growth as those derived with IL-2.

**Conclusions:**

Pulse zoledronate stimulation maximizes the purity, quantity, and quality of expanded Vγ2Vδ2 cells for adoptive immunotherapy but there is no advantage to using IL-15 over IL-2 in our humanized mouse model. Pulse zoledronate stimulation is a simple modification to existing protocols that will enhance the effectiveness of adoptively transferred Vγ2Vδ2 cells by increasing their numbers and anti-tumor activity.

**Electronic supplementary material:**

The online version of this article (doi:10.1186/s40425-017-0209-6) contains supplementary material, which is available to authorized users.

## Background

Cancer is the second leading cause of deaths in the United States and is responsible for 25% of all deaths. Despite advances in our understanding of its causes, treatment had been limited for many tumor types. However, recent successes in cancer immunotherapy are revolutionizing treatment. Intrinsic T cell immunity against tumors can be released by using mAbs to remove inhibition by checkpoint CTLA-4 and PD-1 receptors resulting in responses to many types of tumors. Adoptive immunotherapy with T cells expressing chimeric antigen receptors (CAR) or tumor-reactive αβ T cell antigen receptors (TCRs) have resulted in cures. Yet, significant limitations exist for these therapies. CAR-T therapy is limited to tumors expressing proteins that allow their specific targeting. This has limited their use for solid, non-hematopoietic tumors [1]. Tumor-specific αβ TCRs are difficult to identify and therapy must be individualized for each patient’s MHC. Checkpoint blockade with anti-PD-1 does not work in >75% of lung cancer patients and is even less effective against other tumors, such as colorectal carcinomas, that have few neoantigens due to coding mutations. Although cancer immunotherapy is a breakthrough therapy, additional approaches are needed to realize its full potential.

Treatment with γδ T cells expressing Vγ2Vδ2 TCRs (also termed Vγ9Vδ2 TCRs) is one such therapy that shows promise. In contrast to αβ T cells, the response of human γδ T cells expressing Vγ2Vδ2 TCRs is not MHC restricted [2] but instead require the immunoglobulin superfamily protein, butyrophilin 3A1, that is expressed by all human cells tested [3–7]. Thus, tumor cells and normal cells from all tissues can serve as presenting cells for Vγ2Vδ2 cells. γδ T cells bridge innate and adaptive immunity by using their γδ TCRs in an innate fashion to recognize unconventional ligands associated with cell transformation, infections, and inflammation. γδ T cells expressing Vγ2Vδ2 TCRs are found in primates but not rodents and play important roles in human immunity to microbes and tumors. Vγ2Vδ2 T cells expand to very high numbers during many infections (up to 1 in 2 circulating T cells) and these cells can kill infected cells and tumor cells as well as secrete inflammatory Th1 cytokines, chemokines, and growth factors. Vγ2Vδ2 T cells perform these functions by using their TCRs as pattern recognition receptors that monitor the levels of prenyl pyrophosphates. Prenyl pyrophosphates are essential intermediates in isoprenoid biosynthesis that is required by both microbes and humans. The major endogenous stimulator is isopentenyl pyrophosphate (IPP), an intermediate in the mevalonate pathway, whereas the major microbial stimulator is (*E*)-4-hydroxy-3-methyl-but-2-enyl pyrophosphate (HMBPP), an intermediate in the 2-*C*-methyl-D-erythritol-4-phosphate pathway (reviewed in Ref. [8]).

Vγ2Vδ2 T cells can also use their Vγ2Vδ2 TCRs to directly recognize several malignant B cell lines [9–13] although for the vast majority of tumors there is no direct recognition. In contrast, treatment of tumor cells with aminobisphosphonates allows them to stimulate Vγ2Vδ2 T cells by blocking farnesyl diphosphate synthase (FDPS) (also termed farnesyl pyrophosphate synthase), which leads to the accumulation of its upstream metabolite, IPP. The high levels of IPP can then be detected by Vγ2Vδ2 T cells through their TCRs [14, 15] in a process requiring BTN3A1 [3, 5, 7, 16]. Vγ2Vδ2 T cells also express a variety of NK and SLAM receptors that allow them to recognize and kill certain tumor cell lines [17].

Several cancer immunotherapy treatments have targeted Vγ2Vδ2 T cells either by stimulating Vγ2Vδ2 T cells in vivo [18–22] or by expanding them ex vivo for adoptive transfer [23–31]. Both approaches have had some success in pilot studies treating patients with lymphoma and multiple myeloma as well as non-hematopoietic solid tumors such as prostate and renal cell cancers. However, stimulation of Vγ2Vδ2 T cells in patients or primates with intravenous aminobisphosphonates or prenyl pyrophosphates and IL-2 results in large expansions of Vγ2Vδ2 T cells that wane rapidly on subsequent immunizations [32, 33]. In contrast, adoptive immunotherapy with Vγ2Vδ2 T cells avoids this loss of responsiveness by using frozen lymphocytes obtained by leukapheresis prior to zoledronate therapy. This approach allows patients to be screened to select those that exhibit responsiveness although the majority of patients respond. Very large numbers of Vγ2Vδ2 T cells can be obtained with increases in Vγ2Vδ2 T cell numbers of 400- to 10,000-fold. To date, adoptive γδ immunotherapy has resulted in a durable remission in a patient with metastatic renal cancer [34], a complete remission in a patient with metastatic breast cancer [26], and stable disease in 50% of advanced lung cancer patients [35] with relatively little toxicity.

For most clinical trials and preclinical studies, continuous exposure of PBMC to zoledronate with IL-2 has been used to expand Vγ2Vδ2 T cells where zoledronate is slowly diluted over the course of culture [24–26, 30, 36, 37]. Previously, we found that continuous exposure to zoledronate is toxic to Vγ2Vδ2 T cells [15]. This toxicity results in the suboptimal expansion of Vγ2Vδ2 T cells with relatively narrow dose ranges for all aminobisphosphonates studied. In contrast, a short period of exposure (pulse) of PBMC to aminobisphosphonates results in uniform expansions of Vγ2Vδ2 T cells over a 100-fold concentration range [15].

The cellular toxicity of aminobisphosphonates has been extensively studied and is due to the loss of the FDPS downstream metabolites, farnesyl pyrophosphate (FPP) and geranylgeranyl pyrophosphate (GGPP) and the production of a toxic ATP analog, triphosphoric acid 1-adenosin-5′-yl ester 3-(3-methylbut-3-enyl) ester (ApppI). The loss of FDPS metabolites impairs the transfer of farnesyl or geranylgeranyl chains to the C-termini of small GTPases, such as RAS, RAP, RAB, and RHO, and the γ subunit of G protein-coupled receptors that allows them to anchor to the inner leaflet of membranes and traffic to their proper subcellular locations to function in signal transduction. This loss of normal signaling leads to impaired function and apoptotic cell death and can be partially reversed by the addition of farnesol and/or geranylgeraniol [38–40]. Similarly, the addition of GGPP and IL-18 to purified Vγ2Vδ2 T cells prevents zoledronate toxicity and allows their stimulation [41]. However, these effects were primarily observed with purified Vγ2Vδ2 T cells and these compounds are not approved for clinical use. Unlike prenyl pyrophosphates, zoledronate is not catabolized so its toxicity persists for the culture period as it is slowly diluted. Additionally, the accumulation of IPP leads to the production of the toxic ATP analog, ApppI, that can directly induce apoptosis through inhibition of the mitochondrial adenine nucleotide translocase [42, 43].

Besides bisphosphonate toxicity, an additional factor that could influence the results of adoptive therapy with Vγ2Vδ2 T cells is the γ_C_ growth cytokine used during culture. Present adoptive immunotherapy trials have used IL-2 with zoledronate for Vγ2Vδ2 T cell expansion. However, in mice, CD8 αβ T cells expanded ex vivo using IL-15 [44, 45] or IL-7/IL-15 [46] rather than IL-2, mediate increased tumor immunity. Increases in tumor immunity have been postulated to be due to increases in early/central memory CD8 αβ T cells given that these cells provide better anti-tumor immunity compared to late memory cells [47]. We had earlier shown that IL-15 also supports Vγ2Vδ2 T cell proliferation in response to IPP and, in combination with IL-12, increases IFN-γ production [48]. Thus, expanding Vγ2Vδ2 T cells with IL-15 rather than IL-2 could have benefits but this possibility has not been tested in vivo in humanized mouse models.

In this study, we have compared expanding Vγ2Vδ2 T cells by pulse zoledronate exposure to expanding Vγ2Vδ2 cells by continuous exposure with either IL-2 or IL-15. We find that expanding Vγ2Vδ2 T cells by pulse zoledronate exposure results in higher purity, numbers, and quality of Vγ2Vδ2 T cells. The Vγ2Vδ2 T cells were significantly more effective at mediating tumor immunity in adoptive immunotherapy halting tumor growth and decreasing tumor volume by 50% compared with Vγ2Vδ2 T cells expanded by continuous zoledronate exposure. Pulse zoledronate stimulation similarly improved expansion of Vγ2Vδ2 T cells with IL-15. However, adoptive transfer of Vγ2Vδ2 T cells expanded with IL-15 did not result in improved tumor immunity compared to those expanded with IL-2.

## Methods

### Reagents

FITC-conjugated anti-human Vδ2 TCR (clone B6), allophycocyanin-Cy7-conjugated anti-human CD3 (clone SK7), FITC-conjugated anti-human TCR γδ (clone B1), allophycocyanin-conjugated anti-human Vδ2 mAb (clone B6), FITC- and PE-conjugated anti-human CD3 (clone SP34), PerCP-Cy5.5 anti-human CD27 (clone MT271), PE-Cy7-conjugated anti-human CD28 (clone CD28.2), allophycocyanin-conjugated anti-human CD45RO (clone UCHL1), PE-conjugated anti-human IL-21 (clone 3A3-N2.1), PE-Cy5- and PE-conjugated anti-human CD107a (clone H4A3), PE-conjugated anti-human IFN-γ (clone 4S.B3), and allophycocyanin-conjugated anti-human TNF-α (clone MAB11) antibodies were purchased from BD Biosciences (San Jose, CA). PE-conjugated anti-human CD107a (clone H4A3), PerCP-Cy5.5-conjugated anti-human IL-17 (clone eBio64DEC17), PE-conjugated anti-human granzyme B (clone GB11), PE-conjugated anti-human IL-4 (clone 4D9-8), and PE-conjugated anti-human perforin (clone dG9) antibodies were purchased from eBioscience (San Diego, CA). The PE-conjugated anti-human IL-22 (clone 142928) antibody was purchased from R&D Systems (Minneapolis, MN). Live cells were distinguished from dead cells by staining with Hoechst 33258 or Live/Dead Blue (ThermoFisher Scientific, Waltham, MA). Pamidronate (3 mg/ml) was from Hospira, Inc. (Lake Forest, IL). Zoledronate was provided by Dr. Eric Oldfield.

### Ex vivo expansion of Vγ2Vδ2 T cells

PBMC from random healthy adult donors were obtained by density centrifugation over Ficoll-Hypaque (GE Healthcare Bio-Sciences Corp., Piscataway, NJ) of either random donor leukopaks (12 donors) (obtained from AllCells, LLC., Alameda, CA or the Blood Donor Center at the Dana-Farber Cancer Institute) or fresh blood (donors 2, 3, 4, and 6). Leukopak PBMCs were frozen prior to use whereas PBMC isolated from blood were used directly. Note, we primarily used frozen leukopak PBMC because this is the source of cells for most of the clinical trials assessing the adoptive transfer of Vγ2Vδ2 T cells.

For most experiments, complete media (C-Media) was used and was prepared with 500 ml RPMI 1640 supplemented with 10 ml of 1 M HEPES, 5 ml of 200 mM L-glutamine, 5 ml of 100 mM sodium pyruvate, 5 ml of 50× MEM essential amino acids, 5 ml of 100× MEM non-essential amino acids, 0.5 ml of 55 mM 2-mercaptoethanol, 5 ml of 1000× penicillin/streptomycin (optional), 0.5 ml of 10 mg/ml gentamicin (optional) (all from Thermo Fisher Scientific, Waltham, MA), and 50 ml of fetal calf serum (FCS) and 12 ml of human AB^+^ serum (Gemini Bio-Products, West Sacramento, CA). The media was adjusted to pH ~7.2 with 1.5-2.0 ml of 2 M NaOH prepared with cell culture grade H_2_O and filtered through a 0.2 μm nylon filtration unit. The FCS was prescreened for support of T cell growth by assessing cloning efficiency of human T cell lines or clones. Human serum was prescreened by assessing support of Vγ2Vδ2 T cell expansion in response to HMBPP. A detailed protocol for this media is available on request. OpTmizer^TM^ media was purchased from Thermo Fisher Scientific and used as directed. IL-2 was used at 1000 IU (60 ng/ml or 4 nM) (either as aldesleukin (Chiron, Emeryville, CA) or teceleukin (Roche Holding AG, Basel, Switzerland)). IL-15 was used at 50–100 ng/ml (PeproTech, Inc., Rocky Hill, NJ). For T cell functional assays and for culturing tumor cells, human serum was omitted and 12 ml of FCS was substituted.

For expansions performed in 96-well plates, 1 × 10^5^ PBMCs in 100 μl media was added to equal volumes of media containing varying concentrations of 2× HMBPP or zoledronate per round bottom well. For pulsing, after 4 h the plates were centrifuged and the media with zoledronate removed by flicking and blotting any remaining media on a sterile pad. The cells were washed three times and then 200 μl media was added. On day 3, 100 μl media was removed and replaced with media containing 2× IL-2 or IL-15. For expansions performed in 24-well plates or 75 cm^2^ flasks, PMBC were suspended at 1 × 10^6^ cells/ml in C-media lacking IL-2 with the indicated amount of HMBPP or zoledronate. For pulsing, the cells were incubated for 4 h at 37 °C and 5% CO_2_, washed three times with PBS, and resuspended in C-Media without zoledronate for use. On day 3, 50% of the media was removed and replaced with media containing 2× IL-2 or IL-15. PBMC were incubated for 14 d at 37 °C and 5% CO_2_. Cell growth was monitored by microscope examination and media color. Every 2–4 d, 50% of the media was changed for fresh C-Media (containing IL-2 or IL-15 but without zoledronate or HMBPP) and the cells were split 1:2 depending on cell density. On day 14, the cells were harvested, washed twice with PBS, and counted. Levels of γδ and Vγ2Vδ2 T cells and their differentiation state were assessed by flow cytometric analysis.

### Purification of Vγ2Vδ2 T cells

After ex vivo expansion for 14 d, Vγ2Vδ2 T cells were positively purified using antibody-coated magnetic beads (MACS, Miltenyi Biotec, San Diego, CA). Expanded cells were washed once with PBS, counted, and then 1 × 10^7^ cells were resuspended in 0.1 ml buffer, and reacted with 10 μl of allophycocyanin-conjugated anti-human Vδ2 mAb (clone B6) for 10 min on ice in the dark in ten 2 ml microcentrofuge tubes. Cells were then washed twice with purification buffer, resuspended in 80 μl of buffer, and 20 μl of anti-allophycocyanin magnetic beads added. The cells and beads were incubated on ice for 15 min and then washed twice. 1 × 10^8^ cells were resuspended in 500 μl of purification buffer, and then loaded onto an LS column for positive selection. Retained Vδ2 T cells were washed on the column three times with 3 ml of buffer, removed from the magnetic field, and then eluted, washed, and tested either in vitro for their functional activity or in vivo for their anti-tumor immunity. Cell purify was evaluated by flow cytometry and the Vγ2Vδ2 T cells used were at least 95% Vδ2 cells (most preparations were > 98% and examples of the purity of the Vγ2Vδ2 T cells are shown in Additional file 1: Figure S1). Note that an anti-Vδ2 mAb was used to determine Vγ2Vδ2 T cells after expansion by HMBPP or zoledronate because only γδ T cells expressing Vγ2Vδ2 TCRs respond and expand to prenyl pyrophosphates and aminobisphosphonates [14, 49, 50]. After expansion, all Vδ2 chains are paired with Vγ2 chains because in adult PBMC, a Vδ2 chain is almost always paired with a Vγ2 chain. The converse is not always the case because Vγ2 chains sometimes pair with Vδ1 chains.

### CD107a and intracellular cytotoxic protein and cytokine expression after stimulation of expanded Vγ2Vδ2 T cells

To assess the functional activity of Vγ2Vδ2 T cells expanded under different conditions, the surface mobilization of CD107a, cytotoxic protein levels, and cytokine levels were measured. CD107a surface mobilization was performed as previously described [7, 51]. After 14 d of expansion, Vγ2Vδ2 T cells were either washed twice for use (unpurified cells) or purified using antibody-coated magnetic beads as described above. Human PC-3 prostate cancer cells (ATCC, Manassas, VA) were thawed and cultured in F12 media for 2 d. The PC-3 cells were treated overnight by culture with 200 μM pamidronate. The pamidronate-treated PC-3 cancer cells were washed and used to stimulate unpurified and purified Vγ2Vδ2 T cells. Daudi (ATCC, Manassas, VA) and Raji Burkitt’s lymphoma cell lines were cultured in RPMI 1640, washed, and directly used to stimulate Vγ2Vδ2 T cells. For unpurified Vγ2Vδ2 T cells, E:T ratios were based on the number of Vγ2Vδ2 T cells. Cells were mixed at different E:T ratios (1:1 to 100:1) and cultured in C-media at 37 °C with PE-Cy5- or PE-conjugated anti-CD107a mAbs in the presence of monensin at 4 μl of GolgiStop (BD Biosciences) per 6 ml media. After 4 h, the cells were harvested, washed, stained with allophycocyanin-Cy7- or PE-anti-CD3 and FITC-anti-Vδ2 mAbs, and then staining was assessed by flow cytometry.

To measure cytokine production and cytotoxic protein capabilities, unpurified or purified Vγ2Vδ2 T cells were stimulated with pamidronate-treated PC-3 cancer cells for 4 h in the presence of monensin at 4 μl of GolgiStop (BD Biosciences) per 6 ml media. Alternatively, purified Vγ2Vδ2 T cells were stimulated with ionomycin (2 μg/ml) and PMA (50 ng/ml) (both from Sigma-Aldrich, St. Louis, MO) for 4–6 h in the presence of monensin at 4 μl of GolgiStop (BD Biosciences) per 6 ml media. For flow cytometric analysis, PBMC were first stained with Live/Dead Blue (Invitrogen), to exclude dead cells followed by staining with allophycocyanin-Cy7-conjugated anti-CD3 and FITC-conjugated anti-Vδ2 mAbs. The cells were then washed, fixed, and permeabilized using the Cytofix/Cytoperm Kit (BD Biosciences) and then intracellularly stained with either PE-conjugated anti-IFN-γ, anti-granzyme B, anti-perforin, anti-IL-22, anti-IL-4, allophycocyanin-conjugated anti-TNF-α, or PerCP-Cy5.5-conjugated anti-IL-17a mAbs. Cytokine and cytotoxic protein levels of Vδ2 T cells were assessed by flow cytometry.

### Fluorometric assessment of T lymphocyte antigen specific lysis

Cytotoxicity of expanded Vγ2Vδ2 T cells against tumor cells was determined using fluorometric assessment of T lymphocyte antigen specific lysis (FATAL) [52]. Briefly, PC-3 prostate cancer cells were treated with 200 μM of pamidronate overnight. Pamidronate-treated PC-3 cells were then stained with PKH-26 using a kit (Sigma-Aldrich, St. Louis, MO). PC-3 cells were washed, resuspended in 75 μl of diluent C, mixed with 75 μl of PKH-26 dye (4 × 10^−6^ M), and incubated for 4 min at room temperature. 150 μl of FCS was then added to stop the staining reaction, and the cells washed once in 10 ml of PBS. The cells were then resuspended in 75 μl PBS and mixed with 75 μl of 5 μM carboxyfluorescein diacetate succinimidyl ester (CFSE) dye (Vybrnat CFDA SE Cell Racer Kit, ThermoFisher, Molecular Probes, Eugene, OR). 150 μl of FCS was immediately added to stop the staining reaction. The cells were washed twice with PBS and resuspended for use. 5 × 10^3^ PKH-26/CFSE stained PC-3 cells were then incubated for 5 h with varying numbers of purified Vγ2Vδ2 T cells that had been expanded either by continuous or pulse stimulation. Tumor specific killing by Vγ2Vδ2 T cells was then assessed by flow cytometry. Target % survival was calculated as (mean CFSE^hi^ percent of test well/ mean CFSE^hi^ percent of spontaneous release) × 100. Specific lysis was calculated as 100 − % survival. Lytic units per 1 × 10^7^ T cells were calculated for 20% lysis as 10^7^ / ((5 × 10^3^) × x) where x = E:T ratio corresponding to 20% specific target cell lysis [53].

### Human tumor xenograft and adoptive transfer of purified Vγ2Vδ2 T cells into immunodeficient mice

To assess the anti-tumor activity of adoptively transferred Vγ2Vδ2 T cells, human PC-3 cancer cells were xenotransplanted into immunodeficient mice using a model developed by the Scotet laboratory [54] except that treatment was started one day earlier (day 13 rather than day 14), only purified Vγ2Vδ2 T cells grown for 14 d were used, and treatment was for five weeks rather than four. Note that for these experiments, human Vγ2Vδ2 T cells allogeneic to the tumor were used. Unlike αβ T cells, Vγ2Vδ2 T cells do not exhibit alloreactivity [55]. Therefore, by using purified Vγ2Vδ2 T cells all potential alloreactive responses were avoided.

Female, five-week old NOD.Cg*-Prkdc* ^*scid*^ *Il2rg* ^*tm1Wjl*^/SzJ (NSG) mice were purchased from the Jackson Laboratory (Bar Harbor, ME) and used at 6 weeks of age. For tumor xenotransplantation, 1 × 10^7^ human PC-3 cancer cells were suspended in 200 μl of sterile PBS and s.c. inoculated into the right flank of NSG mice on day 0. Each treatment group consisted of 8 mice. On day 13 (when tumor diameter had reached > 5 mm), mice were injected i.v. with pamidronate (50 μg/kg). The mice weighed 17 to 19 grams and therefore received 0.85 to 0.95 μg of pamidronate. This was followed on day 14 with the i.v. injection of 1 × 10^6^ purified Vγ2Vδ2 T cells that had been expanded either by continuous or pulse zoledronate stimulation with IL-2 or IL-15. For each treatment, Vγ2Vδ2 T cells were purified on day 14 from freshly expanded Vγ2Vδ2 T cells derived from frozen leukopak PBMC from the same donor. These treatments were repeated weekly until week 6. Control mice received only pamidronate treatments. Note that in this model, pamidronate by itself only minimally inhibits the growth of PC-3 tumors [54]. Tumor size was assessed once weekly by external measurement of the longitudinal and transverse tumor diameter using a digital vernier caliper. Tumor volume was calculated using the modified ellipsoidal formula where tumor volume (mm^3^) = (y × *x* ^2^) / 2, where “y” is the longitudinal length and “x” is the transverse width. All experiments involving animals, including their housing and care in pathogen–free conditions.

### Statistical analyses

For statistical analyses, either the unpaired *t* test or the nonparametric Mann–Whitney *U* test was used as indicated with *p* < 0.05 considered statistically significant. Statistical analyses were done in Prism version 6.0 g (GraphPad Software, La Jolla, CA).

## Results

### Pulse zoledronate stimulation improves the purity and number of expanded Vγ2Vδ2 T cells

In our previous study, we found that continuous aminobisphosphonate exposure during culture inhibits the proliferation of Vγ2Vδ2 T cells as well as other γδ and αβ T cells [15]. This toxicity results in the suboptimal expansion of Vγ2Vδ2 T cells over a narrow aminobisphosphonate concentration range whereas pulse exposure results in larger expansions over a wide concentration range [15]. However, in this previous study, the numbers of Vγ2Vδ2 T cells were not determined and only a limited number of donors were used. Also, the activity of Vγ2Vδ2 T cells against tumor cells was not compared for Vγ2Vδ2 T cells grown under the different conditions. Thus, to assess the effect on Vγ2Vδ2 T cells of pulse versus continuous exposure to zoledronate, PBMC were cultured with varying concentrations of zoledronate continuously or for 4 h. On day 14, Vγ2Vδ2 T cells were then measured and their tumor immunity evaluated. Note that PBMC continuously exposed to zoledronate had half of their media changed on day 3 and every 2–4 d thereafter. Thus the full concentration of zoledronate was only present for the first 3 d of culture.

**Fig. 1:**
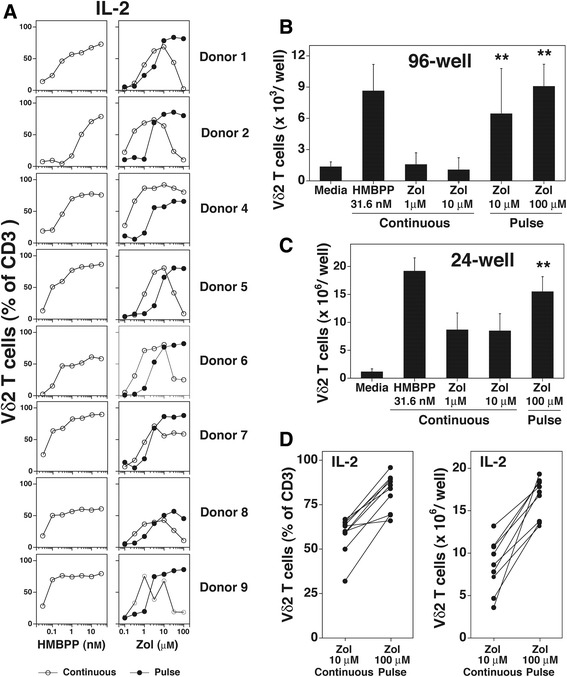
Expanding Vγ2Vδ2 T cells by pulse zoledronate stimulation with IL-2 increased their purity and number compared with continuous stimulation. **a** Expansion of Vγ2Vδ2 T cells in response to varying doses of HMBPP and zoledronate with IL-2. Human Vγ2Vδ2 T cells were expanded ex vivo from PBMC by exposure to HMBPP (*left panels*) or zoledronate (*right panels*). Human PBMC were cultured in 96-well plates either continuously with varying starting concentrations of HMBPP or zoledronate (open circles) or pulsed with zoledronate for 4 h (closed circles) followed by washing twice. IL-2 was added to 1000 IU on day 3. Thereafter, media was changed every 2–3 d depending on cell growth. On day 14, Vγ2Vδ2 T cell numbers were determined by flow cytometric analysis. **b**, **c** Numbers of Vδ2 T cells after 14 d expansion under the indicated conditions with IL-2 for triplicate cultures in either 96-well plates (**b**) (results for eight donors) or 24-well plates (**c**) (results for nine donors). PBMC were cultured for 14 d in the presence of IL-2 at 1 × 10^5^ cells/well for 96-well plates or 1 × 10^6^ cells/well for 24-well plates. **p < 0.01 compared to 10 μM zoledronate continuous stimulation using the Mann–Whitney *U* test. Mean and standard deviation is shown. **d** Comparison of the purity (*left panel*) and number (*right panel*) of Vδ2 T cells expanded by continuous (10 μM) or pulse (100 μM) stimulation with zoledronate with IL-2 in 24-well plates. Each line connects the results for an individual donor. Results for nine donors from (**c**)

Most donors responded to continuous zoledronate exposure with the highest levels of Vγ2Vδ2 T cells between 3–10 μM (Fig. 1a, *right panels*, open circles). For six of the eight donors, higher zoledronate concentrations greatly decreased responsiveness although 2 donors (Donors 4 and 7) responded even at higher concentrations. In contrast, all eight donors responded to pulse zoledronate exposure similarly over a 30-100-fold range from ~10-100 μM (Fig. 1a, *right panels*, closed circles). HMBPP shows no toxic effect on Vγ2Vδ2 T cells even at high concentrations (Fig. 1a, *left panels*).

To assess Vγ2Vδ2 T cell numbers after expansion, zoledronate at 1 μM and 10 μM was chosen for continuous stimulation of PBMC to be consistent with clinical trials. Pulse stimulation with zoledronate was done at 100 μM. Despite achieving similar levels of purity in many cases (Fig. 1a, *right panels*), the number of Vγ2Vδ2 T cells at day 14 expanded by pulse stimulation with zoledronate (100 μM) in 96-well plates averaged 8.6-fold greater than the number of cells derived by continuous stimulation with zoledronate (10 μM) (Fig. 1b, *p* < 0.01). Increases in the number of Vγ2Vδ2 T cells were also noted when PBMC were cultured in 24-well plates with an average of 1.9-fold greater number of cells (Fig. 1c, *p* < 0.01). All donors exhibited increases in purity (mean Vγ2Vδ2 T cells were 58.8% for continuous stimulation versus 80.4% for pulse stimulation, Fig. 1d, *left panel*) and in Vγ2Vδ2 T cell numbers (mean cell numbers were 8.5 × 10^6^ cells for continuous stimulation versus 16.5 × 10^6^ cells for pulse stimulation, Fig. 1d, *right panel*). Increases in cell numbers between donors ranged from 1.4- to 5.4-fold. Thus, exposure of PBMC to zoledronate for a short 4 h period resulted in increased purity and yield of Vγ2Vδ2 T cells compared with continuous zoledronate exposure. These levels are comparable to those achieved with exposure to HMBPP. However, HMBPP has not been used for clinical studies due to a lack of availability of pharmaceutical grade HMBPP and possible patent infringement issues.

### Vγ2Vδ2 T cells expanded by pulse zoledronate stimulation exhibit increased degranulation and perforin expression compared with those expanded by continuous stimulation when stimulated by pamidronate-treated PC-3 cancer cells or Daudi Burkitt’s lymphoma cells

**Fig. 2:**
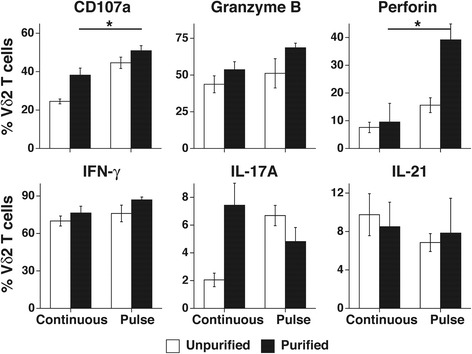
Vγ2Vδ2 T cells expanded by pulse zoledronate stimulation with IL-2 exhibit slightly increased degranulation and expression of granzyme B and perforin compared with those expanded by continuous stimulation when exposed to pamidronate-treated PC-3 cancer cells. Human PBMC were cultured either continuously with zoledronate (5 μM) or pulsed with zoledronate (100 μM) for 4 h and then washed twice before culture with IL-2 (1000 IU/ml). After 14 d, expanded Vγ2Vδ2 T cells were purified by positive selection or left unpurified. PC-3 cancer cells were treated overnight with pamidronate (200 μM) and then washed. Pamidronate-treated PC-3 cells were incubated with unpurified or purified Vγ2Vδ2 T cells for 4 h in duplicate samples followed by surface and intracellular mAb staining. CD107a, IFN-γ, granzyme B, perforin, IL-17A, and IL-21 levels on or in Vγ2Vδ2 T cells were assessed by flow cytometric analysis. Mean ± SD is shown. Representative of two experiments. *p < 0.05 compared to 10 μM zoledronate continuous stimulation using the unpaired *t*-test

To assess the function of expanded Vγ2Vδ2 T cells derived using pulse stimulation, their surface expression of CD107a and intracellular expression of cytotoxic proteins and cytokines were measured in response to TCR stimuli. CD107a (LAMP-1) is a lysosomal membrane protein that is mobilized to the surface when cytotoxic T and NK cells degranulate for killing [51, 56, 57]. When cultured with pamidronate-treated PC-3 prostate cancer cells that have high intracellular levels of IPP, a higher proportion of Vγ2Vδ2 T cells expanded by pulse stimulation expressed CD107a compared with Vγ2Vδ2 cells expanded by continuous stimulation (50.9% versus 38.2% for purified Vγ2Vδ2 T cells, Fig. 2a, *p* < 0.05). Similar results were noted with stimulatory Daudi lymphoma cells with a higher proportion of Vγ2Vδ2 T cells expanded by pulse stimulation expressing CD107a than Vγ2Vδ2 cells expanded by continuous stimulation (31.9% versus 19.5%, *p* = 0.0058, Additional file 1: Figure S2a, b).

Consistent with their high level of CD107a, Vγ2Vδ2 T cells expanded by pulse stimulation had significantly higher proportions expressing perforin compared with Vγ2Vδ2 T cells derived with continuous stimulation. Other cytotoxic proteins and cytokines were expressed at similar levels with Vγ2Vδ2 T cells from both conditions producing high levels of IFN-γ and granzyme B. There were low proportions of Vγ2Vδ2 T cells producing IL-17A (as observed previously in Ref. [58]) and IL-21 (Fig. 2b-f). IL-10 was not produced by expanded Vγ2Vδ2 T cells (data not shown). Activation by the mitogen, ionomycin, with PMA, stimulated IFN-γ and granzyme B to similar very high proportions of Vγ2Vδ2 T cells expanded using either condition (Additional file 1: Figure S2c). Thus, a significantly higher proportion of Vγ2Vδ2 T cells degranulated in response to pamidronate-treated PC-3 prostate cancer cells and Daudi lymphoma cells when derived using pulse stimulation compared with those derived by continuous stimulation.

### Cytotoxicity of Vγ2Vδ2 T cells expanded by pulse zoledronate stimulation against pamidronate-treated PC-3 prostate cancer cells

**Fig. 3:**
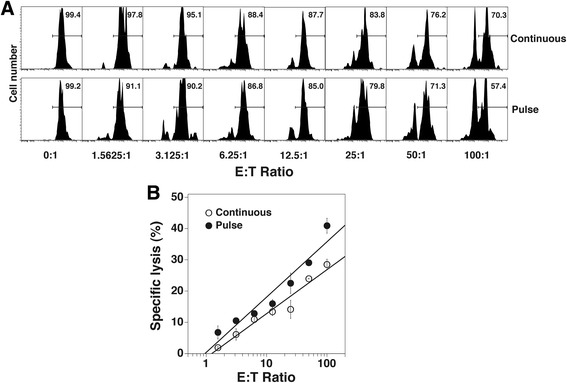
Vγ2Vδ2 T cells expanded by pulse zoledronate stimulation exhibit similar cytotoxicity against pamidronate-treated PC-3 cancer cells compared with Vγ2Vδ2 T cells expanded by continuous stimulation. Vγ2Vδ2 T cells were expanded as described in Fig. 2. Vγ2Vδ2 T cells expanded by continuous or by pulse zoledronate stimulation were then purified for use. PC-3 cancer cells were treated overnight with pamidronate (200 μM) and then washed twice. To assess cytotoxicity, the FATAL assay was used. Pamidronate-treated PC-3 cells were stained with PHK-26 and CFSE and incubated with purified Vγ2Vδ2 T cells for 5 h at various E:T ratios in duplicate. Cells were then fixed with 1% paraformaldehyde followed by flow cytometric analysis. **a** CFSE levels of PC-3 prostate cancer cells incubated with varying numbers of purified Vγ2Vδ2 T cells expanded by continuous or pulse zoledronate stimulation. **b** Cytolytic activity of Vγ2Vδ2 T cells expanded either by continuous or pulse zoledronate stimulation against pamidronate-treated PC-3 cancer cells as determined by the FATAL assay. Representative of two experiments

Cytotoxicity of Vγ2Vδ2 T cells for tumor cells was assessed by fluorometric measurement of tumor cell lysis. In this assay, tumor cells are stained with fluorescent dyes to allow their identification and to evaluate their viability after incubation with cytotoxic T or NK cells [52]. For our experiments, purified Vγ2Vδ2 T cells expanded using either pulse or continuous zoledronate stimulation were mixed at different E:T ratios (1:1 to 100:1) with pamidronate-treated PC-3 tumor cells. Specific killing of PC-3 cells was then assessed by flow cytometric analysis. Vγ2Vδ2 T cells expanded by pulse stimulation killed PC-3 prostate cancer cells more efficiently than those expanded by continuous zoledronate stimulation. Vγ2Vδ2 T cells expanded by pulse stimulation had 155.5 lytic units (for 20% target cell lysis per 1 × 10^7^ T cells) compared with 61.3 lytic units (2.5-fold lower) for Vγ2Vδ2 T cells expanded by continuous stimulation (Fig. 3a, b). Thus, consistent with the CD107a expression results, Vγ2Vδ2 T cells expanded by pulse stimulation exhibited higher levels of cytotoxicity for tumor cells than those expanded using continuous zoledronate stimulation.

### Adoptive transfer of Vγ2Vδ2 T cells expanded using pulse zoledronate stimulation controlled PC-3 tumor growth in NSG mice

**Fig. 4:**
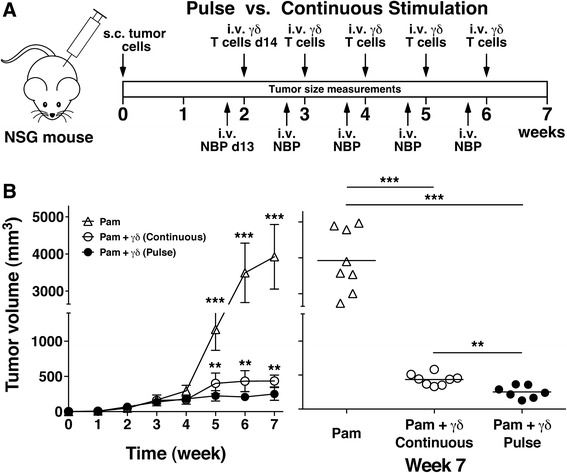
Adoptive transfer of Vγ2Vδ2 T cells expanded by pulse zoledronate stimulation in combination with pamidronate controlled PC-3 prostate tumor growth in NSG mice. **a** Schema of treatment protocol used to evaluate the anti-tumor efficacy of Vγ2Vδ2 T cells. Immunodeficient NSG mice were s.c. inoculated with human PC-3 prostate cancer cells on day 0. On day 13, pamidronate (50 μg/kg) was given i.v. On day 14, 1 × 10^6^ purified Vγ2Vδ2 T cells expanded using either continuous or pulse zoledronate stimulation were inoculated i.v. Treatments were repeated weekly until week 6. Longitudinal and transverse diameters of the tumors were measured weekly. **b**, *left panel* Vγ2Vδ2 T cells stimulated by pulse zoledronate exposure exhibit significantly better anti-tumor immunity compared with those expanded by continuous zoledronate exposure. Mean PC-3 tumor volume ± SD is shown for 7–8 mice per group treated with either pamidronate alone (open triangles), pamidronate with purified Vγ2Vδ2 T cells derived by continuous zoledronate stimulation (open circles), or pamidronate with purified Vγ2Vδ2 T cells derived by pulse zoledronate stimulation (closed circles). **p < 0.01, ***p < 0.001 compared with tumor volume of mice treated with Vγ2Vδ2 T cells derived by pulse zoledronate stimulation using the Mann–Whitney *U* test. *Right panel*, Tumor volume at week 7 of individual mice treated with pamidronate alone (open triangles), pamidronate with Vγ2Vδ2 T cells derived by continuous zoledronate stimulation (open circles), or pamidronate with Vγ2Vδ2 T cells derived by pulse zoledronate stimulation (closed circles). Bars represent mean values. **p < 0.01, ***p < 0.001 using the Mann–Whitney *U* test

To assess the in vivo anti-tumor activity of Vγ2Vδ2 T cells expanded under the two conditions, a humanized immunodeficient mouse model developed by the Scotet laboratory was used [54]. In this model, human PC-3 prostate cancer cells are xenotransplanted into the flanks of highly immunodeficient NSG mice followed 2 weeks later by 4 weekly treatment with the aminobisphosphonate, pamidronate, and adoptively transferred Vγ2Vδ2 T cells (schema shown in Fig. 4a). The only modifications to their protocol that were made were that mice were treated with pamidronate on day 13 instead of day 14 and that freshly purified Vγ2Vδ2 T cells (>98% purity, Additional file 1: Figure S1) from 14 day cultures were used for each adoptive transfer rather than unpurified Vγ2Vδ2 T cells from cultures of varying ages. The use of purified Vγ2Vδ2 T cells allowed us to directly compare the anti-tumor activity of Vγ2Vδ2 T cells expanded under the two conditions using identical numbers of transferred T cells. Vγ2Vδ2 T cells were expanded from frozen leukopak PBMC derived from a single donor to more closely approximate present clinical treatment protocols.

Adoptive transfer of Vγ2Vδ2 T cells expanded under either condition greatly slowed tumor growth with large decreases in tumor volume (Fig. 4b) and tumor diameter (Additional file 1: Figure S3b) compared with pamidronate treatment alone. These results are identical to those reported by Santolaria et al. [54]. Pamidronate treatment alone had no effect on tumor growth whereas mice treated with pamidronate followed by Vγ2Vδ2 T cells derived using continuous zoledronate stimulation slowed tumor growth with slight increases in tumor volume and diameter. In contrast, tumor growth stopped in mice treated with Vγ2Vδ2 T cells expanded using pulse zoledronate stimulation with significantly lower tumor volumes (Fig. 4b, *p* < 0.01) and tumor diameters (Additional file 1: Figure S3b, *p* < 0.01) compared with treatment with Vγ2Vδ2 T cells expanded using continuous zoledronate stimulation. Tumor volume was reduced by 42% at week 7 (Fig. 4b, *right panel*) and averaged 46% reductions for weeks 5–7. Thus, while adoptive transfer of Vγ2Vδ2 T cells expanded under either condition greatly slowed tumor growth, tumors in mice treated with Vγ2Vδ2 T cells expanded using pulse stimulation were about half the size of tumors in mice treated with Vγ2Vδ2 T cells expanded using continuous stimulation. This better tumor control is consistent with the increased cytotoxicity of these cells in vitro.

### Pulse zoledronate stimulation improves the purity and yield of Vγ2Vδ2 T cells cultured with IL-15

**Fig. 5:**
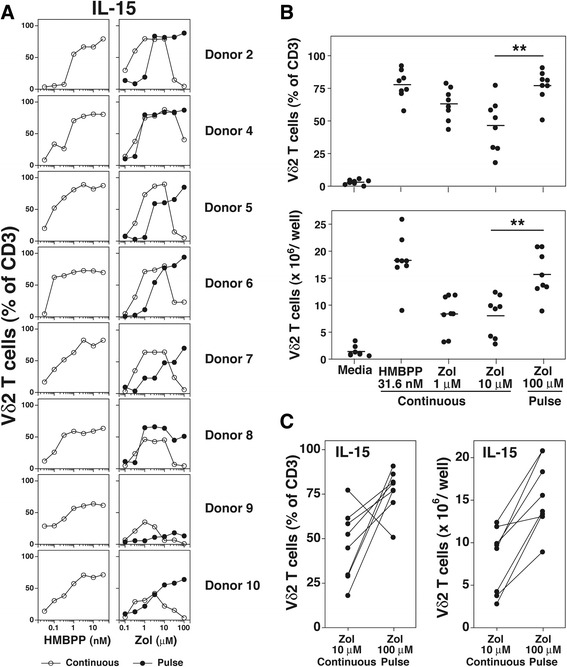
Expanding Vγ2Vδ2 T cells by pulse zoledronate stimulation with IL-15 increased their purity and numbers compared with continuous stimulation. **a** Expansion of Vγ2Vδ2 T cells in response to varying doses of HMBPP and zoledronate with IL-15. Human Vγ2Vδ2 T cells were expanded ex vivo from PBMC by exposure to HMBPP (*left panels*) or zoledronate (*right panels*). Human PBMC were cultured in 96-well plates either continuously with varying starting concentrations of HMBPP or zoledronate (open circles) or pulsed with varying concentrations of zoledronate for 4 h (closed circles) followed by washing twice. IL-15 was added to 100 ng/ml on day 3. Thereafter, media was changed every 2–3 d depending on cell growth. On day 14, Vγ2Vδ2 T cell numbers were determined by flow cytometric analysis. Results for 8 donors are shown. **b**, *upper panel* Purity of Vδ2 T cells under the indicated conditions with IL-15 (100 ng/ml) after 14-day culture in 24-well plates. Each point represents one donor (*n =* 8). ** *p* = 0.003, Mann–Whitney *U* test. *Lower panel*, Number of Vδ2 T cells under the indicated conditions with IL-15 (100 ng/ml) after 14-day culture in 24-well plates. Each point represents one donor (*n =* 8). ** *p* = 0.007, Mann–Whitney *U* test. **c** Comparison of the purity (*left panel*) and number (*right panel*) of Vδ2 T cells expanded by continuous (10 μM) or pulse (100 μM) stimulation with zoledronate with IL-15 in 24-well plates. Each line connects the results for an individual donor from (**b**)

IL-15 is a cytokine supporting T cell growth that is a member of the common γ_C_ cytokine family that also includes IL-2, IL-7, and IL-21. Tumor-reactive murine CD8 αβ T cells expanded ex vivo using IL-15 [44, 45] or IL-7/IL-15 [46] mediate increased tumor immunity in vivo as compared to cells grown in IL-2. However, comparable studies have not been reported for Vγ2Vδ2 T cells. Therefore, we assessed whether pulse zoledronate stimulation would also improve expansion of Vγ2Vδ2 T cells when IL-15 was used. Similar to stimulation with IL-2, Vγ2Vδ2 T cells expanded using continuous zoledronate stimulation in 96-well plates showed maximal expansion between 1–10 μM with higher concentrations greatly decreasing expansion (Fig. 5a, *right panels*). In contrast, expansion of Vγ2Vδ2 T cells using pulse zoledronate stimulation showed expansion over a broader range of up to 100-fold (Donors 4 and 8). Maximal purity levels were similar between the various conditions and to levels observed with HMBPP stimulation (except for Donor 9). Direct comparison for individual donors showed similar results for IL-2 and IL-15 when evaluated in parallel (Additional file 1: Figure S4).

Both the purity and number of Vγ2Vδ2 T cells was higher with pulse zoledronate stimulation compared with continuous stimulation with levels similar to those observed with HMBPP (Fig. 5b). All donors except one, exhibited increases in purity (Fig. 5c, *left panel*) and in Vγ2Vδ2 T cell numbers (Fig. 5c, *right panel*) with pulse zoledronate stimulation. Increases in cell numbers ranged from 1.1- to 4.8-fold, averaging 2.4-fold for the 8 donors. These findings were consistent with those observed with Vγ2Vδ2 T cell expansion with IL-2.

### IL-15 preserves higher proportions of early/central memory subsets of Vγ2Vδ2 T cells

**Fig. 6:**
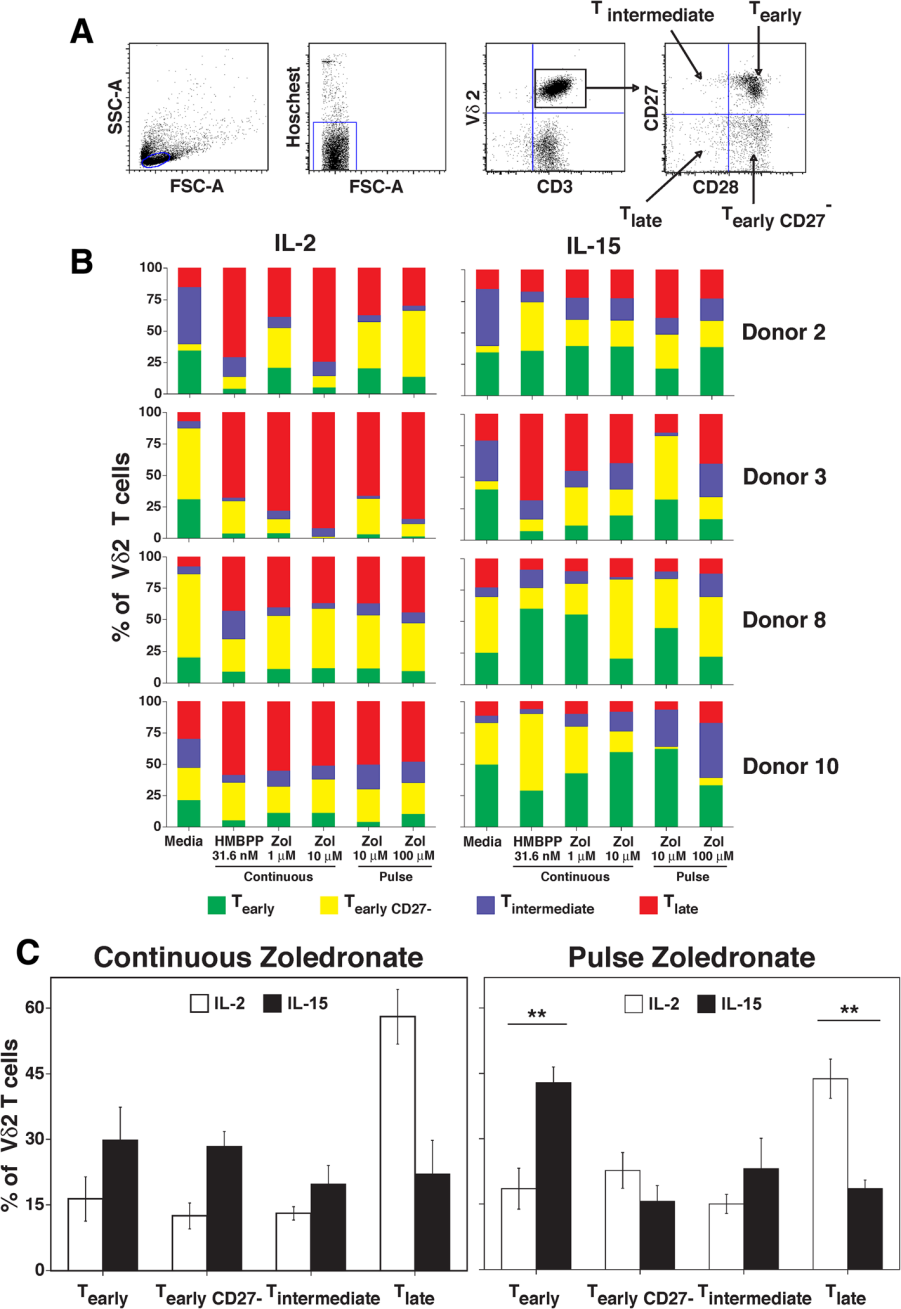
IL-15 sustained higher proportions of early/central memory subsets among expanded Vγ2Vδ2 T cells compared to IL-2. **a** Gating strategy for delineating memory subsets of Vγ2Vδ2 T cells from blood. Memory status of Vγ2Vδ2 T cells was assessed by expression of CD27 and CD28. CD28^+^/CD27^+^ and CD28^+^/CD27^−^ cells are early/central memory cells that express CD45RO. CD28^−^/CD27^+^ cells are intermediate memory cells. CD28^−^/CD27^−^ cells are late memory cells that express CD45RA. **b** Proportions of human Vγ2Vδ2 T cell memory subsets after expansion with the indicated concentrations of HMBPP or zoledronate by continuous and pulse stimulation and in the presence of IL-2 (*left panels*) or IL-15 (*right panels*) for 14 days. Data for four donors is shown. **c**, *left panel* Comparison of the proportion of memory subsets of Vγ2Vδ2 T cells expanded by continuous zoledronate stimulation (10 μM) with IL-2 or IL-15. Mean ± SEM for 4 individuals. *Right panel*, Comparison of the proportion of memory subsets of Vγ2Vδ2 T cells expanded by pulse zoledronate stimulation (100 μM) with IL-2 or IL-15. Mean ± SEM for 4 individuals. ** *p* < 0.01 using the unpaired *t*-test

Previous studies with αβ T cells have shown that IL-15 specifically sustains the early/central memory subsets of CD8 αβ T cells [59, 60] and in combination with IL-7 maintain stem αβ T cells [61]. Early/central memory CD8 αβ T cells confer stronger anti-tumor immunity upon adoptive immunotherapy compared with late/effector T cells in mouse models [47, 62]. To determine the effect of IL-15 on Vγ2Vδ2 T cells, we compared the proportion of memory subsets in the presence of either IL-2 or IL-15 following zoledronate or HMBPP stimulation. CD28 and CD27 were used as phenotypic markers for different Vγ2Vδ2 T cell memory subsets because these receptors distinguish early/central memory (CD28^+^ CD27^+/−^) from intermediate (CD28^−^ CD27^+^) or late/effector memory (CD28^−^ CD27^−^) cells (data not shown and [63]). The gating strategy is shown for Vγ2Vδ2 T cells from PBMC (Fig. 6a). Note that in adults, almost all Vγ2Vδ2 T cells are memory cells with the naive population being very small (<2%).

When Vγ2Vδ2 T cells were expanded by zoledronate or by HMBPP stimulation in the presence of IL-15, the majority of Vγ2Vδ2 T cells were early/central memory cells (colored green (CD28^+^ CD27^+^) or yellow (CD28^+^ CD27^−^) in Fig. 6b, *right panels*) whereas the majority of Vγ2Vδ2 T cells expanded in the presence of IL-2 were late/effector memory cells (colored red (CD28^−^ CD27^−^) or blue (CD28^−^ CD27^+^) in Fig. 6b, *left panels*). There were significantly more early/central memory T cells when Vγ2Vδ2 T cells were expanded by pulse zoledronate stimulation in IL-15 and more late/effector memory T cells with pulse zoledronate stimulation in IL-2 (Fig. 6c, *right panel*). Similar results were noted with continuous stimulation (Fig. 6c, *left panel*). Therefore, IL-15 maintained a higher proportion of early/central memory subsets of Vγ2Vδ2 T cells than IL-2.

### Vγ2Vδ2 T cells expanded using pulse zoledronate stimulation with IL-15 express CD107a, cytotoxic proteins, and cytokines in similar proportions to those expanded with IL-2

To assess the effect of IL-15 on Vγ2Vδ2 T cell function, the expression of CD107a, cytotoxic proteins, and cytokines were measured in Vγ2Vδ2 T cells expanded by pulse zoledronate stimulation with IL-15 in comparison to Vγ2Vδ2 T cells expanded with IL-2. PC-3 cancer cells were treated with pamidronate to increase intracellular IPP levels. The treated PC-3 cells were then used to stimulate purified Vγ2Vδ2 T cells that were derived using pulse zoledronate stimulation with either IL-2 or IL-15. CD107a, granzyme B, perforin, IFN-γ, TNF-α, and IL-4 expression was then assessed by flow cytometry. No significant differences were noted in the proportion of Vγ2Vδ2 T cells expressing CD107a, granzyme B, perforin, IFN-γ, and TNF-α between Vγ2Vδ2 T cells expanded with IL-15 to those expanded with IL-2 (Additional file 1: Figure S5). There were somewhat higher proportions of Vγ2Vδ2 T cells producing IL-4 when expanded with IL-15 but these cells constituted a small population (<6%) and are of uncertain significance in vivo. Thus, Vγ2Vδ2 T cells expanded with IL-15 have similar functional capabilities to those expanded with IL-2.

### Adoptive transfer of Vγ2Vδ2 T cells expanded using pulse stimulation with IL-15 controlled PC-3 tumor growth in NSG mice similarly to Vγ2Vδ2 T cells expanded with IL-2

**Fig. 7:**
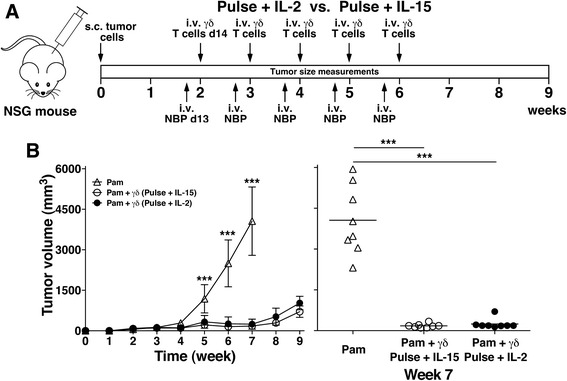
Adoptive transfer of Vγ2Vδ2 T cells expanded by pulse zoledronate stimulation with IL-15 in combination with pamidronate controlled PC-3 prostate tumor growth in NSG mice similarly to Vγ2Vδ2 T cells expanded by pulse zoledronate stimulation with IL-2. **a** Schema of treatment protocol used to evaluate the anti-tumor efficacy of Vγ2Vδ2 T cells. Immunodeficient NSG mice were s.c. inoculated with human PC-3 prostate cancer cells on day 0. On day 13, pamidronate (50 μg/kg) was given i.v. On day 14, 1 × 10^6^ purified Vγ2Vδ2 T cells expanded using pulse zoledronate stimulation with either IL-15 or IL-2 were inoculated i.v. Treatments were repeated weekly until week 6. Longitudinal and transverse diameters of the tumors were measured once weekly until week 9. **b**, *left panel* Vγ2Vδ2 T cells stimulated by pulse zoledronate exposure with IL-15 showed similar anti-tumor immunity compared with those expanded with IL-2. Mean PC-3 tumor volume ± SD is shown for 7–8 mice per group treated with either pamidronate alone (open triangles), pamidronate with purified Vγ2Vδ2 T cells derived by pulse zoledronate stimulation with IL-15 (open circles), or pamidronate with purified Vγ2Vδ2 T cells derived by pulse zoledronate stimulation with IL-2 (closed circles). ***p < 0.001 compared with mean tumor volume of mice treated with Vγ2Vδ2 T cells derived by pulse zoledronate stimulation with IL-2 using the Mann–Whitney *U* test. *Right panel*, Tumor volume at week 7 of individual mice treated with pamidronate alone (open triangles), pamidronate with Vγ2Vδ2 T cells derived by pulse zoledronate stimulation with IL-15 (open circles), or pamidronate with Vγ2Vδ2 T cells derived by pulse zoledronate stimulation with IL-2 (closed circles). Bars represent mean values. ***p < 0.001 using the Mann–Whitney *U* test

To assess the effect of IL-15 on anti-tumor activity, Vγ2Vδ2 T cells expanded using pulse zoledronate stimulation with either IL-15 or IL-2 were adoptively transferred into NSG mice bearing PC-3 prostate tumors (Fig. 7a). Both Vγ2Vδ2 T cells expanded with IL-15 or with IL-2 efficiently controlled PC-3 tumors stopping their growth but not eliminating the tumors. There were no significant differences in tumor volume (Fig. 7b) or tumor diameter (Additional file 1: Figure S6) when Vγ2Vδ2 T cells expanded with IL-15 (open circles) were compared to those expanded with IL-2 (closed circles). Moreover, at week 8, two weeks after adoptive transfer of Vγ2Vδ2 T cells had stopped, tumor growth resumed in both groups without significant differences albeit at a slower rate of growth than PC-3 tumors treated with pamidronate alone. Therefore, expansion of Vγ2Vδ2 T cells with IL-15 did not improve immunity against prostate tumors in our humanized mouse model. This result is consistent with the absence of significant differences in the in vitro function of Vγ2Vδ2 T cells expanded with IL-15 compared to those expanded with IL-2.

### Vγ2Vδ2 T cells stimulated by pulse zoledronate expand to similar levels using OpTmizer™ media manufactured under cGMP and with large scale cultures

**Fig. 8:**
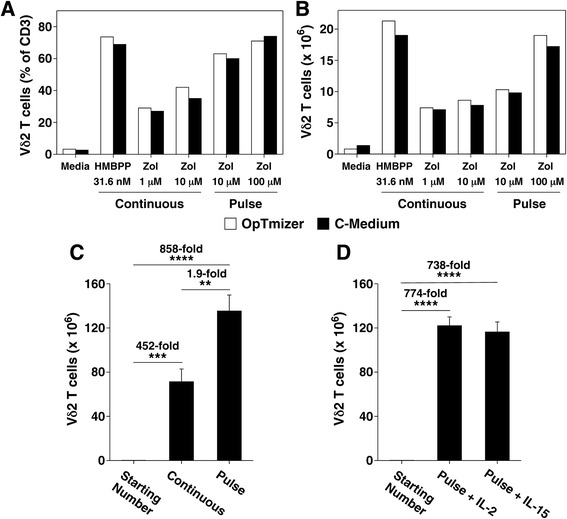
Vγ2Vδ2 T cells stimulated by pulse zoledronate expand to similar levels using commercial cGMP grade OpTmizer™ media and with larger-scale cultures. **a**, **b** Similar purity (**a**) and yield (**b**) of Vγ2Vδ2 T cells expanded by pulse zoledronate stimulation in cGMP grade OpTimizer™ culture media as compared with supplemented RPMI 1640 C-media. Vγ2Vδ2 T cells from frozen leukopak PBMC were expanded by pulse zoledronate stimulation with IL-2 for 14 d in either OpTimizer™ culture media or the supplemented RPMI 1640 C-media used in this study. Vγ2Vδ2 T cell numbers were determined by flow cytometric analysis. Representative of two experiments. **c**, **d** Expansion of Vγ2Vδ2 T cells by pulse zoledronate culture under larger-scale cultures yielded similar enhancements in yield as noted with small-scale cultures. For larger-scale expansion of Vγ2Vδ2 T cells, leucopak PBMCs were stimulated by either continuous (5 μM) or pulse zoledronate (100 μM) exposure (**c**) as detailed in Fig. 1 with IL-2 in C-Media in 75 cm^2^ cell culture flasks in triplicate cultures. For (**d**), Vγ2Vδ2 T cells were expanded by pulse zoledronate stimulation with either IL-2 or IL-15 in C-Media in 75 cm^2^ cell culture flasks in triplicate cultures. After 14 days, Vγ2Vδ2 T cell numbers were determined by flow cytometric analysis. Starting number of Vγ2Vδ2 T cells was 0.158 × 10^6^ for each group. Mean ± SD is shown. **p < 0.01, ***p < 0.001, ****p < 0.0001 using the unpaired *t*-test

To determine if pulse zoledronate stimulation could improve current practices in clinical trials, we examined whether similar results could be obtained using commercial media used for T cell expansions that is produced under current good manufacturing practices (cGMP) and with larger scale expansions. To meet regulatory requirements, culture media used for clinical trials must meet cGMP standards. Therefore, we compared the enriched RPMI 1640 media used in our experiments (termed C-media) with OpTmizer™ media, a media meeting cGMP standards. Expansion of Vγ2Vδ2 T cells in OpTmizer™ media (open bars) resulted in comparable levels of Vγ2Vδ2 T cell purity (Fig. 8a) and numbers (Fig. 8b) to those achieved using C-media (solid bars). Consistent with experiments using C-media, expansion of Vγ2Vδ2 T cells in OpTmizer™ media using pulse zoledronate stimulation resulted in higher purity (71% vs. 42% Vγ2Vδ2 T cells) and cell numbers (19.0 × 10^6^ versus 8.6 × 10^6^ Vγ2Vδ2 T cells, 2.2-fold higher) compared with expansion using continuous stimulation (Fig. 8a, b).

Similar increases in cell numbers were noted with larger scale expansions. Pulse zoledronate stimulation of thawed leukopak PBMC in flasks resulted in an 858-fold increase in Vγ2Vδ2 T cell numbers by day 14 (1.58 × 10^5^ cells increasing to 1.35 × 10^8^ cells, Fig. 8c). This expansion was 1.9-fold higher than that achieved by continuous zoledronate stimulation. Consistent with our experiments in 24-well plates, expansion of Vγ2Vδ2 T cells using pulse zoledronate stimulation with IL-2 in flasks gave similar cell numbers when IL-15 was used (774-fold with IL-2 versus 738-fold with IL-15, Fig. 8d). These results demonstrate that pulse zoledronate stimulation resulted in increased Vγ2Vδ2 T cell purity and numbers with cGMP media and larger scale expansions. Similar benefits are likely, not only for preclinical in vitro studies and humanized mouse models, but also for clinical trials employing adoptive transfer of Vγ2Vδ2 T cells.

## Discussion

Adoptive immunotherapy with Vγ2Vδ2 T cells for the treatment of a variety of cancers has proven to be safe with 203 patients treated with few severe toxicities [64, 65]. Vγ2Vδ2 T cells can be expanded from most cancer patients and these patients can be easily identified by testing in small-scale cultures. Patients treated with Vγ2Vδ2 T cells have had partial or complete remissions or stable disease but most patients progress. Therefore, discovering ways to increase the effectiveness of Vγ2Vδ2 T cell therapy is required to realize its full potential. In this report, we show that expanding Vγ2Vδ2 T cells by pulsing PBMC with zoledronate increases their purity and numbers compared with continuous zoledronate stimulation by decreasing zoledronate toxicity to Vγ2Vδ2 T cells. Vγ2Vδ2 T cells expanded by pulse zoledronate stimulation expressed higher levels of perforin and a larger proportion degranulated when exposed to stimulatory tumor cells. These Vγ2Vδ2 T cells killed tumor cells more strongly with a 2.5-fold increase in cytolytic activity compared with those expanded using continuous zoledronate stimulation. Consistent with the increase in cytotoxicity, adoptive transfer of Vγ2Vδ2 T cells expanded by pulse stimulation halted tumor growth in vivo, reducing tumor volume by half compared with equal numbers of Vγ2Vδ2 T cells expanded by continuous stimulation. Thus, limiting zoledronate exposure by pulsing increased both the quantity of Vγ2Vδ2 T cells and their effectiveness in tumor immunity.

One potential limitation of this study was our use of normal donors. However, we and others have found that the requirements for ex vivo expansion of Vγ2Vδ2 T cells from cancer patients are identical to those observed with normal donors [33, 36]. The main difference between the normal donors and patients is that a higher proportion of cancer patients fail to respond if they have advanced metastatic disease, are elderly, or have been treated with chemotherapy or intravenous aminobisphosphonates [26, 28, 36, 66]. Previously using a modified 24 h pulse of zoledronate, the main predictor for poor Vγ2Vδ2 T cell expansion in 18 breast cancer patients was blood Vγ2Vδ2 T cell levels less than 1% [33]. The 83% of patients with Vγ2Vδ2 T cell levels greater than 1% had similar expansions compared with normal donors regardless of the cancer stage. However, because a higher proportion of breast cancer patients had levels less than 1% compared with normal donors, there were higher numbers of poor zoledronate responders. Higher proportions of patients with metastatic cancers also had Vγ2Vδ2 T cell levels of less than 1% and poor expansions whereas those with higher levels were able to expand normally [26]. Cancer patients with normal blood Vγ2Vδ2 T cell levels including those with breast cancer, prostate cancer, lung cancer, colorectal cancer, and hepatocellular carcinoma expanded similarly to normal donors whereas those with low levels did not [36, 67–70]. Because the requirements for expansion of Vγ2Vδ2 T cells do not appear to differ between normal donors and cancer patients, pulse zoledronate stimulation will give comparable improvements in expansion with cancer patients whose Vγ2Vδ2 T cells are greater than 1% as we have previously shown [33]. Pulse zoledronate stimulation could also help improve expansion in patients with lower Vγ2Vδ2 levels given that pulse exposure of dendritic cells to zoledronate helped to restore Vγ2Vδ2 expansion in tumor patients that were non- or weakly responsive to continuous bromohydrin pyrophosphate stimulation [68].

Given that zoledronate targets the same enzyme, FDPS, in all cells, why does limiting the period of zoledronate exposure improve expansion of Vγ2Vδ2 T cells? This is due to differences in the rate of uptake of zoledronate and other aminobisphosphonates between different cell types. Aminobisphosphonates enter cells through fluid-phase endocytosis because they cannot passively diffuse through cell membranes due to the negative charges on their phosphonate moieties [71, 72]. Metabolically active cells such as monocytes/macrophages and tumor cells have increased rates of fluid-phase endocytosis compared with resting Vγ2Vδ2 T cells. Indeed, in PBMC, only monocytes efficiently internalize aminobisphosphonates with little or no uptake by other cells including T cells [73]. Consistent with this selective uptake, there are large increases in IPP/DMAPP in monocytes after zoledronate pulsing but only small increases in T cells [73] and monocytes are the primary APC for aminobisphosphonate stimulation of Vγ2Vδ2 T cells in PBMC [74]. Also, unlike resting T cells in PBMC, highly metabolically active Vγ2Vδ2 T cell clones do not proliferate when continuously exposed to risedronate (although they do release TNF-α) presumably due to their uptake of risedronate [15]. Risedronate also inhibits the proliferation of αβ and other γδ T cells in response to IL-2 or PHA [15]. Thus, pulsing of PBMC with zoledronate allows for its uptake into monocytes but not into resting Vγ2Vδ2 T cells, thereby reducing alterations in cell signaling and direct toxicity due to ApppI.

The short period of aminobisphosphonate exposure during the in vitro pulse period is similar to what is observed in blood upon their intravenous administration. Aminobisphosphonate have very short half-lives in plasma (1–2 hours) due to their renal excretion and deposition into bone (where the half-life of alendronate is estimated to be 10 years) [75, 76]. Even a short exposure to zoledronate can result in prolonged elevations in IPP in cells. Once bound into the dimethylallyl pyrophosphate site of FDPS, aminobisphosphonates function as nearly irreversible inhibitors because IPP binding to its site in FDPS induces a conformational change locking them in place by closing the binding site [77]. This property allows a brief exposure to zoledronate to effect prolonged inhibition of FDPS enzymatic activity. For example, increases in IPP levels are noted for 10 days (with peak levels at day 7) in intraperitoneal monocytes after treatment of mice with zoledronate [78] and for up to 7 days in monocytes after in vitro exposure [79]. In the NSG mouse model used in this report, human PC-3 tumor cells isolated from mice treated with pamidronate stimulated CD107a expression on Vγ2Vδ2 T cells above background for up to 5 days after treatment [54]. These results show that given sufficient uptake, a brief exposure to zoledronate can cause prolonged elevations in cellular IPP levels.

If continuous zoledronate exposure is toxic for T cells, why are repeated intravenous treatments with pamidronate required for effective PC-3 tumor control in NSG mice? As noted above, aminobisphosphonates given intravenously are rapidly cleared from the blood within a few hours. Tumor cells preferentially take up aminobisphosphonates because their high rates of proliferation and metabolic activity necessitates constant uptake of nutrients by fluid phase endocytosis. In this respect, aminobisphosphonates are acting similar to traditional chemotherapeutic agents that target tumor cells because of their rapid proliferation by disrupting DNA synthesis, mitosis, nucleotide synthesis, or other cellular processes. After cell entry, aminobisphosphonates are released into the cytoplasm where they blocks FDPS resulting in IPP/ApppI accumulation, This accumulation can be sensed by Vγ2Vδ2 T cells through their TCRs resulting in activation. However, in the PC-3 NSG mouse model, PC-3 tumor cells only remain stimulatory for up to 5 days [54]. Therefore, repeated pamidronate treatments were required to make PC-3 cells stimulate Vγ2Vδ2 T cells through their TCRs.

The downside of intravenous aminobisphosphonates and prenyl pyrophosphates or their analogs is that they anergize and/or delete Vγ2Vδ2 T cells [32, 33, 80, 81]. This is likely due to stimulation of Vγ2Vδ2 T cells by non-professional APCs that either lack the appropriate costimulatory signals, activate too strongly, or are in the wrong location for the effective stimulation and maintenance of memory T cells. Vγ2Vδ2 T cells present around the time of aminobisphosphonate infusion, including those previously transferred, will be anergized and/or deleted. To avoid this, either zoledronate therapy is avoided or else PBMC are isolated from patients by leukapheresis and stored in liquid nitrogen prior to starting intravenous zoledronate therapy [26, 27, 34]. Similar anergy and deletion of Vγ2Vδ2 T cells were noted with intravenous bromohydrin pyrophosphate [32] or zoledronate in monkeys (unpublished observations) or in breast cancer patients receiving zoledronate [33]. Thawed PBMC harvested prior to zoledronate treatments can then be used for ex vivo expansion of Vγ2Vδ2 T cells for adoptive transfer as we have done in this study. Thus, for effective anti-tumor immunity it is insufficient to treat only with aminobisphosphonates, new populations of Vγ2Vδ2 T cells are also needed explaining the requirement for repeated cycles of pamidronate and Vγ2Vδ2 T cells for control of PC-3 tumor growth in NSG mice [this study and Ref. 54] or alendronate and Vγ2Vδ2 T cells for control of MeWo melanoma and PancTu1 pancreatic adenocarcinoma tumors in SCID mice [82]. With weekly treatments, PC-3 tumor growth was completely halted for the duration of treatment with tumor growth resuming two weeks after completion of therapy (Fig. 4 and 7).

Vγ2Vδ2 T cells expanded with IL-15 and pulse zoledronate stimulation showed identical tumor immunity upon in vivo testing to those expanded with IL-2. Despite preserving a higher proportion of early/central memory subsets that mediate improved tumor immunity in mice [45, 83], transfer of Vγ2Vδ2 T cells expanded with IL-15 inhibited PC-3 tumor growth in NSG mice identically to equal numbers of Vγ2Vδ2 T cells expanded with IL-2 (Fig. 7). This finding may reflect the limitations of the humanized mouse model used in this study. In the PC-3 model, there are relatively short intervals between the infusions of Vγ2Vδ2 T cells that may limit the impact of any increase in the persistence of the Vγ2Vδ2 T cells. Moreover, in the clinical trials with the best outcomes, intravenous zoledronate was given either on the day of each cell infusion [27, 34] or one day prior to and on the day of cell infusion [26]. These approaches are similar to the one used in our mouse model. In this model, treatment by adoptive transfer of Vγ2Vδ2 T cells without pamidronate or giving pamidronate only once prior to the first cell infusion does not inhibit PC-3 tumor growth. Only when pamidronate is given prior to each Vγ2Vδ2 T cell infusion is PC-3 tumor growth significantly controlled [54]. As discussed above, intravenous aminobisphosphonates or prenyl pyrophosphates rapidly leads to anergy and/or deletion of Vγ2Vδ2 T cells which would limit the advantage of increasing their persistence.

Our results are consistent with studies on human CD8 αβ T cells. Melanoma-specific CD8 αβ T cells expand to a similar degree in high dose IL-2 (300 IU/ml) or IL-15 and produce equal amounts of IFN-γ. Similar results were observed with CAR-T cells expressing gp100-reactive TCRs and with naive or adult T cells stimulated with anti-CD3 [84]. In vitro expansion is also similar when either high dose IL-2 (1000 IU/ml), IL-15, or a combination of the two is used for human CD8 αβ T cells specific for melanoma, influenza, Epstein-Barr virus, and cytomegalovirus [85]. Importantly, specific lysis of melanomas and IFN-γ release in response to melanoma cells is similar [85]. Cytomegalovirus-specific CD8 αβ T cells also expand similarly with IL-2 or IL-15 and exhibit similar levels of cytotoxicity, IFN-γ and TNF-α production [86]. These results and ours differ from the in vitro results using IL-15 with Vγ2Vδ2 T cells in a recent study [87]. In this study, however, relatively low dose IL-2 (100 IU/ml) was used, some of the experiments used pure Vγ2Vδ2 T cells omitting accessory cells or used both IL-2 and IL-15, and the cells were not tested for their in vivo tumor activity [87].

Our results differ from those reported for murine models. In the Pmel-1 murine melanoma model, the majority of Pmel-1 CD8 αβ T cells expressing transgenic TCRs specific for the self/tumor specific peptide, gp100_25–33_ are naive when stimulated in vitro with the gp100_25–33_ peptide and either IL-2 or IL-15 [44, 83]. Stimulation differentiates these naive T cells into enriched populations of effector (IL-2) or central (IL-15) memory T cells. Pmel-1 T cells expanded in IL-15 exhibit enhanced tumor immunity compared with those expanded in IL-2 [83]. Similarly, only P14 TCR transgenic T cells stimulated with the gp33-44 peptide with IL-15 mediate tumor immunity to B16_gp33_ melanoma cells [45]. T effector generated from naive precursors exhibit more anti-tumor immunity than those generated from memory cells [88] and naive and early effector tumor-specific murine T cells exhibit more anti-tumor immunity than effector T cells [62].

This use of naive T cells for generating effector T cells for transfer is a significant difference between human Vγ2Vδ2 T cells and murine TCR transgenic T cells that may explain the difference in IL-15 effects. Essentially all adult Vγ2Vδ2 T cells are memory cells with only a very small proportion (1.6%) with naive phenotypes (data not shown and Ref. [89]). Therefore, most expanded Vγ2Vδ2 T cells are derived from memory Vγ2Vδ2 T cells rather than naive cells. We speculate that this fact diminishes the effectiveness of IL-15 because the Vγ2Vδ2 cells being stimulated are already memory cells rather than differentiating from naive cells to memory under the influence of IL-15 whereupon the cells would retain more early/central memory capabilities.

Thus, we would conclude that expansion of Vγ2Vδ2 T cells in IL-15 could help persistence in vivo but without zoledronate treatment to render the tumor cells stimulatory to Vγ2Vδ2 T cells, they would have limited ability to control tumor growth. If zoledronate or another aminobisphosphonate is given, then the Vγ2Vδ2 T cells that are present will likely be rapidly anergized and/or deleted limiting the benefits of increased Vγ2Vδ2 T cell survival. Although in vivo administration of IL-15 could show benefits by promoting memory T cell survival [59, 90, 91], we predict that using IL-15 instead of IL-2 for ex vivo Vγ2Vδ2 T cell expansion is unlikely to show benefits.

To treat patients, cell expansions must be performed in media produced under cGMP. To confirm that pulse zoledronate stimulation would show similar benefits, we compared the results obtained with the enriched RPMI 1640 media used in our experiments (termed C-media) with OpTmizer™ media, a media meeting cGMP standards. OpTmizer™ media gives higher numbers of Vγ2Vδ2 T cell after stimulation with continuous zoledronate stimulation compared with conventional RPMI 1640 or AIM-V™ media with 1% human serum [92]. Clinical trials in Japan have used ALys203 - γδ or ALys505 enriched media (based on Iscove’s DMEM media) with 2-10% autologous plasma [24, 25, 27–30, 37]. We have also used another enriched media, Yssel’s media, that is also based on Iscove’s DMEM [93] with 10% human serum, for expansion with a 24 h zoledronate pulse [33]. PBMC were incubated with 5 μM zoledronate for 24 h followed by removal of spent media and addition of fresh media such that the zoledronate concentration was reduced to 0.625 μM. This modified pulse method also gave high rates of expansion with Vγ2Vδ2 T cells purity of 97% of T cells at day 10. In the present study, we found that expansion of Vγ2Vδ2 T cells in C-media with FCS gave comparable expansions to those obtained in OpTmizer™ media confirming the utility of this method of expansion for use with cGMP compliant media.

C-media could be a cost-effective option for preclinical studies as it is based on inexpensive RPMI 1640 yet gives comparable results to OpTmizer™ media. In preclinical studies testing Vγ2Vδ2 T cells in vitro or in vivo in humanized mouse models, suboptimal expansion of Vγ2Vδ2 T cells is commonly reported and could be due to continuous zoledronate stimulation in non-enriched media. Poor quality Vγ2Vδ2 T cells could skew the results in these studies. For example, testing novel aminobisphosphonates by Vγ2Vδ2 T cell expansion using continuous stimulation gave difficult to interpret results due to toxicity that likely could be avoided by pulsing [94]. Toxicity differences likely explain the varying levels of maximal Vγ2Vδ2 T cell expansion observed with different aminobisphosphonates [15, 95].

Besides limiting exposure to zoledronate, there may be other ways to improve ex vivo expansion of Vγ2Vδ2 T cells to generate cells with better anti-tumor immunity. The mechanistic target of rapamycin (mTOR) kinase regulates myriad cellular functions including metabolism, protein synthesis, growth, survival, apoptosis, and autophagy [96, 97]. Inhibition of mTOR with rapamycin enhances memory CD8 αβ T cell generation and function [98, 99] through downregulation of T-bet and upregulation of Eomesodermin [98, 100]. Rapamycin enhances anti-tumor responses in murine tumor models [100, 101]. Moreover, a short treatment with rapamycin increases the activity of human CD8 αβ T cells upon adoptive transfer into NSG mice bearing human melanomas [91]. Consistent with these increases in tumor activity, although long term rapamycin treatment delays ex vivo expansion, the resulting Vγ2Vδ2 T cells express higher levels of CD25, less CCR5, and are more cytotoxic [102]. Therefore, a short treatment of Vγ2Vδ2 T cells with rapamycin prior to adoptive transfer could enhance their anti-tumor activity without delaying expansion.

Another possible way to improve anti-tumor immunity of Vγ2Vδ2 T cells is to alter the cytokines used during expansion. IL-18 added to IL-2 increases Vγ2Vδ2 T cell numbers by 2.7-fold after zoledronate stimulation of PBMC from breast cancer patients and increases the production of IFN-γ and TNF-α [33]. IL-18 with IL-2 also increases the number of CD56^+^CD11c^+^ NK-like cells [33, 103]. Given that a type of pulse zoledronate exposure was used, similar increases in numbers and function will likely occur with the pulse zoledronate stimulation described in this study. Although Vγ2Vδ2 T cells grown in IL-15 did not mediate better anti-tumor immunity, synergistic effects between IL-15 and a second γ_C_ cytokine, IL-21, have been reported [104–106]. In addition to enhancing T cell proliferation, IL-21 maintains expression of CD28 [105, 106]. Unlike IL-2, IL-21 suppresses differentiation of CD8 αβ T cells to effector memory cells [107]. When given in vivo in mice, IL-21 boosts anti-tumor immunity [104, 108]. By itself, IL-21 does not support efficient expansion of Vγ2Vδ2 T cells and drives them to acquire characteristics of follicular homing T cells that provide B cell help [109, 110]. However, the combination of IL-21 and IL-2 supports expansion while also increasing the ability of Vγ2Vδ2 T cells to kill tumor cells and produce inflammatory cytokines [111]. Thus, expanding Vγ2Vδ2 T cells with pulse zoledronate stimulation and either IL-18/IL-2, IL-21/IL-15, or IL-21/IL-2 could increase their anti-tumor immunity.

Besides using different cytokines during ex vivo expansion, alternatives exist to using zoledronate for stimulation. Prenyl pyrophosphates, such as HMBPP, are not toxic for Vγ2Vδ2 T cells and two analogs, bromohydrin pyrophosphate [23] and 2-methyl-3-butenyl-1-pyrophosphate [27, 112], have been used for Vγ2Vδ2 T cell expansions for clinical trials. However, these compounds are patented investigational drugs and not approved for general clinical use. Pharmaceutical grade HMBPP is not available and there are possible patent issues with its use. Monoclonal antibodies reactive with CD2 and CD3 [113, 114], CD3 [115], and the γδ TCR [116, 117] or artificial K562 APCs expressing membrane bound IL-15 with IL-2 and IL-21 [118] have been used to expand γδ T cells that can then be purified for use either as a mixed γδ TCR population or as purified Vγ2Vδ2 T cells. However, the mAbs are not currently approved for clinical use. Finally, more potent aminobisphosphonates can be used for expansion of Vγ2Vδ2 T cells by pulse stimulation including lipophilic pyridinium bisphosphonates [119] lipophilic zoledronate derivatives [120], and bisphosphonate prodrugs [121].

The results of our in vivo studies point to a critical role for the tumor and the tumor microenvironment in preventing Vγ2Vδ2 T cells from completely eliminating tumors. Despite halting tumor growth for five weeks, adoptive transfer of Vγ2Vδ2 T cells did not cure established PC-3 tumors in NSG mice. Tumor growth resumed two weeks after cessation of therapy (Fig. 7b). This is similar to the results in clinical trials with Vγ2Vδ2 T cells where stable disease is the most common positive outcome. For example, adoptive transfer of Vγ2Vδ2 T cells stabilized advanced non-small cell lung cancer with one patient alive at 4.5 years (5-years survival is normally only 3%) and >50% of patients alive at ~2 years (2-years survival with present treatments is <8%) [25, 35]. Because we transferred purified Vγ2Vδ2 T cells, there were no human T_reg_ or other human αβ T cells. Also, NSG mice have no murine B cells, T cells, or functional NK cells but they do have myeloid-derived suppressor cells. Thus, the tumor and the tumor microenvironment likely prevent Vγ2Vδ2 T cells from completely eliminating tumors suggesting that treatments targeting these factors would increase tumor immunity.

Tumors protect against immune attack through a variety of mechanisms [122, 123]. One likely mechanism inhibiting Vγ2Vδ2 T cells is tumor expression of ligands for checkpoint receptors. Effector T cells express a variety of inhibitory and costimulatory receptors that control their function. Inhibitory receptors play important roles in preventing autoimmune responses. These same receptors also inhibit anti-tumor responses. Blocking the action CTLA-4 and PD-1 receptors or their ligands by mAbs is effective in treating a number of different tumors including melanoma, NSCLC, bladder cancer, renal cancer, cancers with mismatch repair enzyme deficiency, and Hogkin’s lymphoma [124]. Other inhibitory receptors or their ligands have been identified, including BTLA (CD272), LAG-3 (CD223), TIM-3 (CD366), TIGIT, VISTA, B7-H3 (CD276), and B7-H4, that have been found to increase the effectiveness of anti-PD-1 and anti-CTLA-4 mAbs in mouse models. Loss of activity of human CAR-T cells against tumor cells in NSG mice or humans was associated with expression of multiple inhibitory receptors including PD-1, BTLA, LAG3, TIM3, and 2B4 [125, 126]. Checkpoint receptor blockade with anti-PD-1 or anti-PD-L1 mAbs enhances murine CAR-T tumor immunity in mouse models [127–129] and human CAR-T immunity against human lung cancer xenografts in NSG mice [130]. Vγ2Vδ2 T cells can express PD-1 [131] and BTLA [132] that inhibit their functions. We have found that PD-1, CTLA-4, LAG-3, and TIM-3 are all upregulated after stimulation of Vγ2Vδ2 T cells with zoledronate such that 60-95% of Vγ2Vδ2 T cells expressed these receptors by day 7 (data not shown). We hypothesize that a similar upregulation of inhibitory receptors occurs in patients treated with zoledronate and Vγ2Vδ2 T cells because zoledronate treatment would stimulate the Vγ2Vδ2 T cells. This would limit tumor immunity provided by the transferred Vγ2Vδ2 T cells.

Besides checkpoint blockade, tumor cells and surrounding stromal and immune regulatory cells also produce various metabolites and cytokines that inhibit effector T cell function. Indoleamine 2,3-dioxygenase (IDO) isoforms are rate limiting enzymes in a pathway that catabolizes L-tryptophan to kynurenine and other toxic metabolites that inhibit effector T cells [133] while also depleting L-tryptophan (reviewed in [134, 135]). Tumor expression of IDO also promotes the differentiation and activation of Foxp3^+^ regulatory T cells [reviewed in 135] that serve to recruit myeloid derived suppressor cells [136]. IDO inhibitors, such as 1-methyl-D-trytophan (indoximod) and epacadostat, enhance anti-tumor immunity either alone or in combination with mAbs against CTLA-4 or PD-1 in murine models [137–139] or in studies with CD19-CAR T cells in humanized mouse models [140] and are presently in clinical trials.

Tumors and mesenchymal stem cells also produce prostaglandin E_2_ (PGE_2_) that inhibits the function of Vγ2Vδ2 T cells, decreasing their cytotoxicity and production of inflammatory cytokines [66, 141–143]. PGE_2_ is produced through the action of COX1 and COX2 enzymes and COX2 upregulated in a some cancers [144]. PGE_2_ protects tumors in mice from T cell dependent growth control and blocking PGE_2_ production with aspirin synergizes with anti-PD-1 to control tumor growth [145]. Thus, blocking COX1/COX2 to inhibit PGE_2_ production could enhance the anti-tumor immunity of Vγ2Vδ2 T cells.

Adoptive immunotherapy with Vγ2Vδ2 T cells could be an important addition to present cancer treatments. Although safe, Vγ2Vδ2 T cell therapy has thus far had low response rates. This therapy has many potential advantages. Vγ2Vδ2 T cell are stimulated by a wide array of tumors because recognition does not require neoantigens or tumor-specific proteins or MHC expression but instead is dependent on alterations in isoprenoid metabolism and expression of BTN3A1. As discussed above, aminobisphosphonate treatment is similar to traditional chemotherapeutic agents because it targets tumor cells because of their rapid proliferation and high metabolic activity. But here the result is elevations of IPP that stimulate Vγ2Vδ2 T cells through their TCRs. Treatment with Vγ2Vδ2 T cells could be used for a variety of solid tumors or for those patients that fail other immunotherapies. Unlike αβ CAR-T cells, no preconditioning of patients with chemotherapy or radiation therapy is required. To improve efficacy, Vγ2Vδ2 T cell therapy could be combined with checkpoint blockade to enhance the anti-tumor immunity of transferred Vγ2Vδ2 T cells while also releasing intrinsic neoantigen-specific αβ T cells. Depending on the tumor, mAbs targeting different checkpoint receptors could help improve the effectiveness of Vγ2Vδ2 T cells. Drugs blocking IDO or PGE_2_ synthesis would likely also add to their effectiveness. Drug treatments or the use of different cytokines could improve ex vivo Vγ2Vδ2 T cell expansion. To realize the full potential of Vγ2Vδ2 T cell treatments, we need to determine what combination of therapies results in effective tumor control.

## Conclusions

Here we describe a modification to ex vivo expansion of Vγ2Vδ2 T cells that improves adoptive immunotherapy for prostate cancer in a humanized mice model. Pulse zoledronate stimulation maximizes the purity, quantity, and quality of expanded Vγ2Vδ2 cells. This simple modification to existing protocols will enhance the effectiveness of adoptively transferred Vγ2Vδ2 T cells.

## Additional file

Additional file 1: Figure S1. Purity of Vγ2Vδ2 T cells after positive magnetic bead separation. Vγ2Vδ2 T cells expanded by either continuous or pulse zoledronate stimulation for 14 d and then purified by magnetic bead separation. **Figure S2.** Expansion of Vγ2Vδ2 T cells by pulse zoledronate stimulation increased degranulation as measured by the expression of CD107a in response to the stimulatory Burkitt’s lymphoma cell line, Daudi. **Figure S3.** Adoptive transfer of Vγ2Vδ2 T cells expanded by pulse zoledronate stimulation in combination with pamidronate controlled PC-3 prostate tumor growth in NSG mice; tumor diameter data for Fig. 4. **Figure S4.** Comparison of the expansion of Vγ2Vδ2 T cells in response to varying doses of HMBPP and zoledronate with IL-2 or IL-15. **Figure S5.** Functional capabilities of Vγ2Vδ2 T cells expanded by pulse zoledronate stimulation with IL-15 are similar to those expanded with IL-2. **Figure S6.** Adoptive transfer of Vγ2Vδ2 T cells expanded by pulse zoledronate stimulation with IL-15 in combination with pamidronate controlled PC-3 prostate tumor growth in NSG mice similarly to Vγ2Vδ2 T cells expanded by pulse zoledronate stimulation with IL-2; tumor diameter data for Fig. 7. (PDF 947 kb)

## Abbreviations

ApppI: Triphosphoric acid 1-adenosin-5′-yl ester 3-(3-methylbut-3-enyl) ester
CAR: Chimeric antigen receptor
cGMP: Current good manufacturing practices
C-Media: Complete media
DMAPP: Dimethylallyl pyrophosphate (diphosphate)
FATAL: Fluorometric assessment of T lymphocyte antigen specific lysis
FDPS: Farnesyl diphosphate (pyrophosphate) synthase
FPP: Farnesyl pyrophosphate
GGPP: Geranylgeranyl pyrophosphate
HMBPP: (*E*)-4-hydroxy-3-methyl-but-2-enyl pyrophosphate (diphosphate)
IDO: Indoleamine 2,3-dioxygenase
IPP: Isopentenyl pyrophosphate (diphosphate)
mTOR: Mechanistic (mammalian) target of rapamycin
TCR: T cell antigen receptor
