# Supplementary material for: Enhancing adoptive cancer immunotherapy with Vγ2Vδ2 T cells through pulse zoledronate stimulation
Source: J Immunother Cancer. 2017 Feb 21;5:9. doi: 10.1186/s40425-017-0209-6 (PMC5319075; doi:10.1186/s40425-017-0209-6)
Supplement: Additional file 1: Figure S1. — Purity of Vγ2Vδ2 T cells after positive magnetic bead separation. Vγ2Vδ2 T cells expanded by either continuous or pulse zoledronate stimulation for 14 d and then purified by magnetic bead separation. Figure S2. Expansion of Vγ2Vδ2 T cells by pulse zoledronate stimulation increased degranulation as measured by the expression of CD107a in response to the stimulatory Burkitt’s lymphoma cell line, Daudi. Figure S3. Adoptive transfer of Vγ2Vδ2 T cells expanded by pulse zoledronate stimulation in combination with pamidronate controlled PC-3 prostate tumor growth in NSG mice; tumor diameter data for Fig. 4. Figure S4. Comparison of the expansion of Vγ2Vδ2 T cells in response to varying doses of HMBPP and zoledronate with IL-2 or IL-15. Figure S5. Functional capabilities of Vγ2Vδ2 T cells expanded by pulse zoledronate stimulation with IL-15 are similar to those expanded with IL-2. Figure S6. Adoptive transfer of Vγ2Vδ2 T cells expanded by pulse zoledronate stimulation with IL-15 in combination with pamidronate controlled PC-3 prostate tumor growth in NSG mice similarly to Vγ2Vδ2 T cells expanded by pulse zoledronate stimulation with IL-2; tumor diameter data for Fig. 7. (PDF 947 kb) [file 40425_2017_209_MOESM1_ESM.pdf]

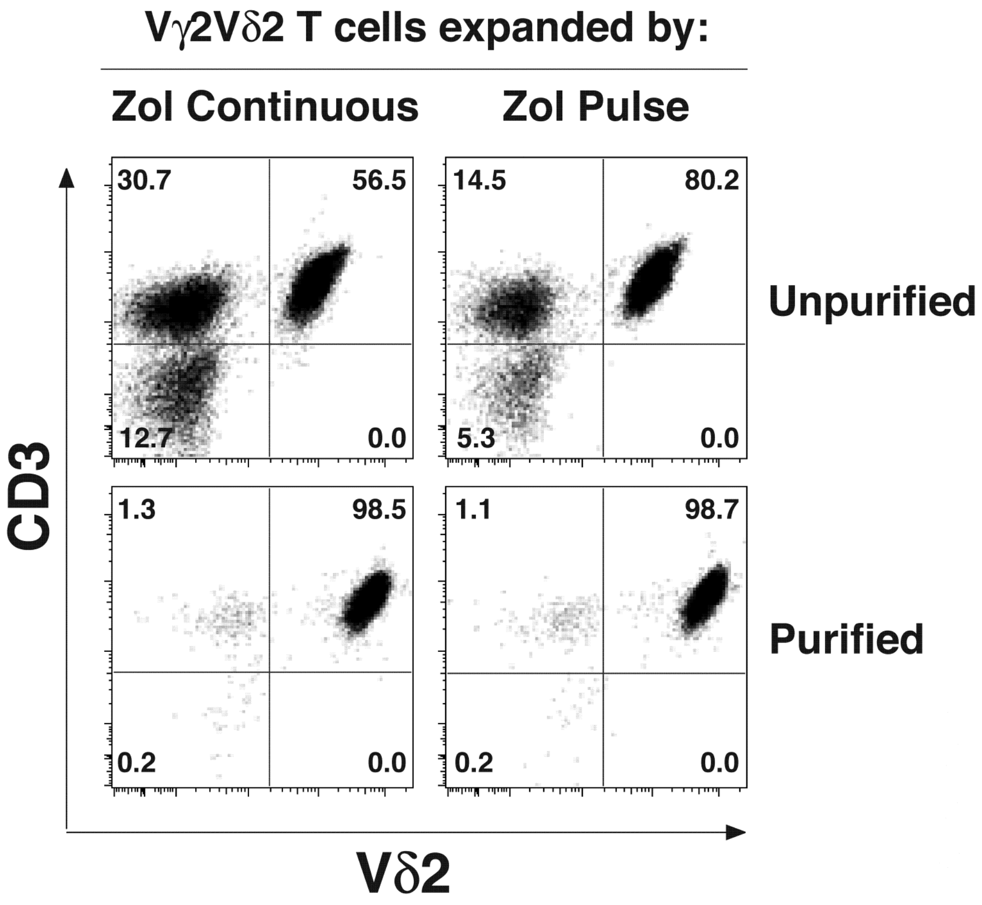


**Figure S1.** Purity of Vγ2Vδ2 T cells after positive magnetic bead separation. Vγ2Vδ2 T cells expanded by either continuous or pulse zoledronate stimulation for 14 d and then purified by magnetic bead separation. Expanded Vγ2Vδ2 T cells (*top panels*) were washed, resuspended to $1 \times 10^7$ cell/ml, and reacted with APC-conjugated anti-human Vδ2 mAb (clone B6) for 20 min. Cells were then washed twice with purification buffer and anti-APC magnetic beads were added. After incubation on ice for 15 min, the cells were washed twice, resuspended in 500 µl of purification buffer, and loaded onto LS columns for positive selection. Purified Vδ2 T cells were removed from the magnet, eluted, washed, and then counted for use for adoptive transfer or other functional assays. Cell purity was evaluated by flow cytometry (*bottom panels*) and was at least > 95% for use.

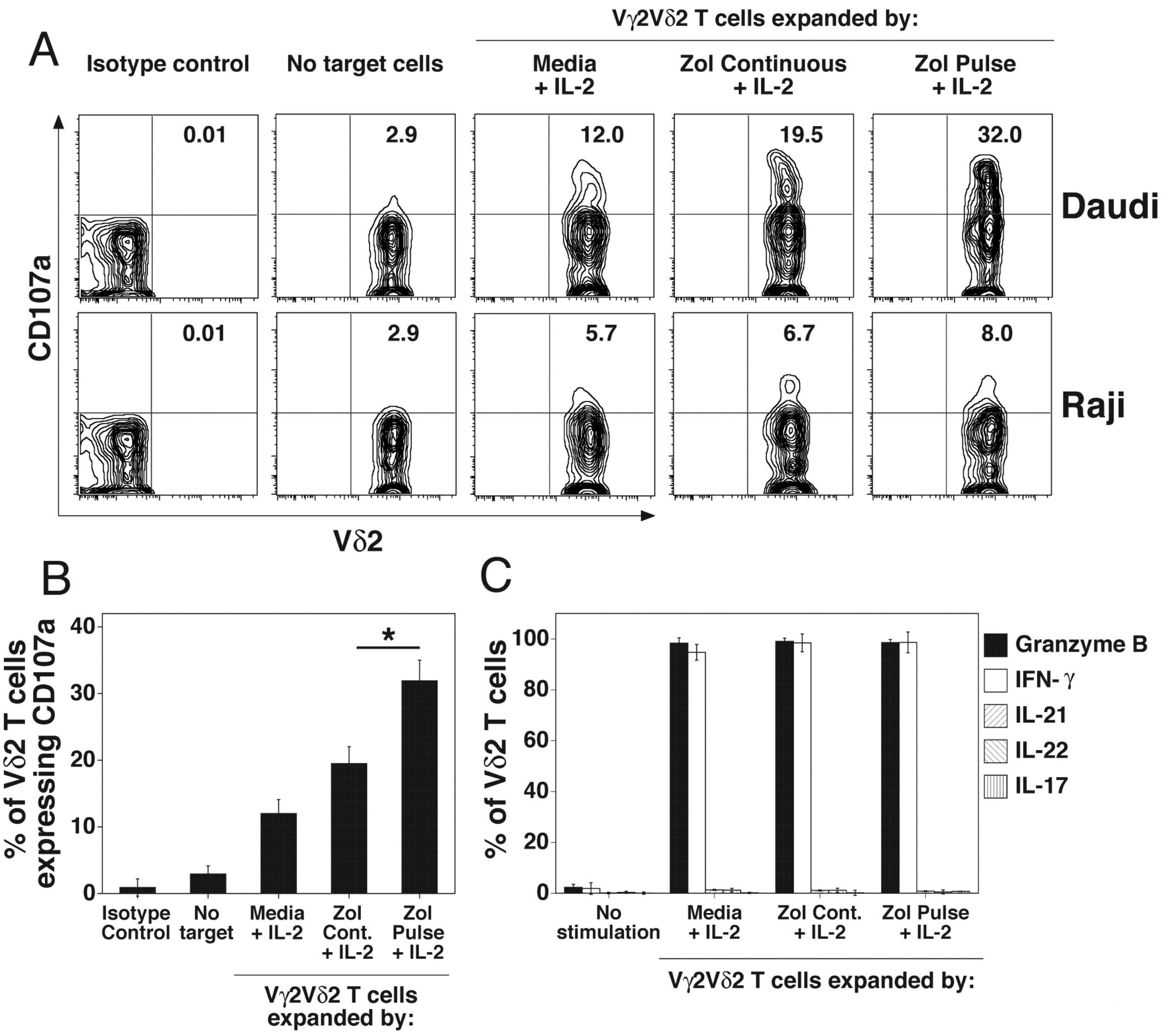


**Figure S2.** Expansion of Vγ2Vδ2 T cells by pulse zoledronate stimulation increased degranulation as measured by the expression of CD107a in response to the stimulatory Burkitt's lymphoma cell line, Daudi. **a** Increased expression of CD107a by Vγ2Vδ2 T cells expanded by pulse zoledronate stimulation when exposed to the stimulatory Daudi cell line. Vγ2Vδ2 T cells were expanded from PBMCs by either continuous or pulse zoledronate stimulation for 14 d. Vγ2Vδ2 T cell numbers were assessed by flow cytometric analysis. Unpurified Vγ2Vδ2 T cells were then mixed with Daudi or Raji Burkitt's lymphoma cells at 10:1 (E:T) ratio for 4 h in the presence of monensin and PE-Cy7-anti-CD107a. The cultures were then washed and stained with PE-anti-CD3 and FITC-anti-Vδ2 mAbs and analyzed by flow cytometry. **b** Mean % of Vδ2 T cells expressing CD107 after culturing with Daudi cells (n = 3 experiments). ***$p$ = 0.0058 using the unpaired *t*-test. **c** Similar levels of IFN-γ and granzyme B in Vγ2Vδ2 T cells expanded either by continuous or pulse zoledronate stimulation or by culturing in IL-2 after ionomicin/PMA stimulation for 4 h. Levels were determined by intracellular staining and flow cytometric analysis.

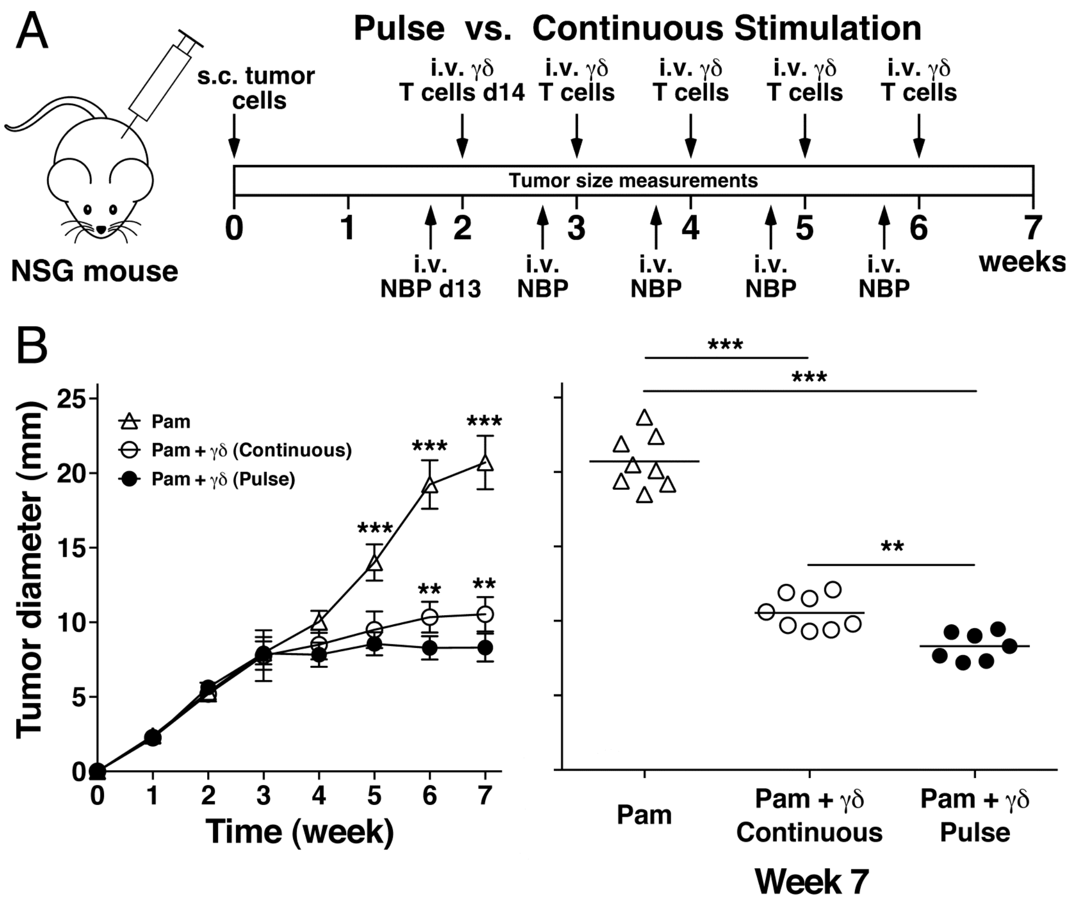


**Figure S3.** Adoptive transfer of Vγ2Vδ2 T cells expanded by pulse zoledronate stimulation in combination with pamidronate controlled PC-3 prostate tumor growth in NSG mice; tumor diameter data for Fig. 4. **a** Schema of treatment protocol used to evaluate the anti-tumor efficacy of Vγ2Vδ2 T cells. Immunodeficient NSG mice were s.c. inoculated with human PC-3 prostate cancer cells on day 0. On day 13, pamidronate (50 μg/kg) was given i.v. On day 14, $1 \times 10^6$ purified Vγ2Vδ2 T cells expanded either by continuous or by pulse zoledronate stimulation were inoculated i.v. Treatments were repeated weekly until week 6. Longitudinal and transverse diameters of the tumors were measured weekly. **b**, *left panel* Vγ2Vδ2 T cells stimulated by pulse zoledronate exposure exhibit significantly better anti-tumor immunity compared with those expanded by continuous zoledronate exposure. Mean PC-3 tumor diameter ± SD is shown for 7-8 mice per group treated with either pamidronate alone (open triangles), pamidronate with purified Vγ2Vδ2 T cells derived by continuous zoledronate stimulation (open circles), or pamidronate with purified Vγ2Vδ2 T cells derived by pulse zoledronate stimulation (closed circles). **$p < 0.01$, ***$p < 0.001$ compared with tumor volume of mice treated with Vγ2Vδ2 T cells derived by pulse zoledronate stimulation using the Mann-Whitney *U* test. *Right panel*, Tumor volume at week 7 of individual mice treated with pamidronate alone (open triangles), pamidronate with Vγ2Vδ2 T cells derived by continuous zoledronate stimulation (open circles), or pamidronate with Vγ2Vδ2 T cells derived by pulse zoledronate stimulation (closed circles). Bars represent mean values. **$p < 0.01$, ***$p < 0.001$ using the Mann-Whitney *U* test.

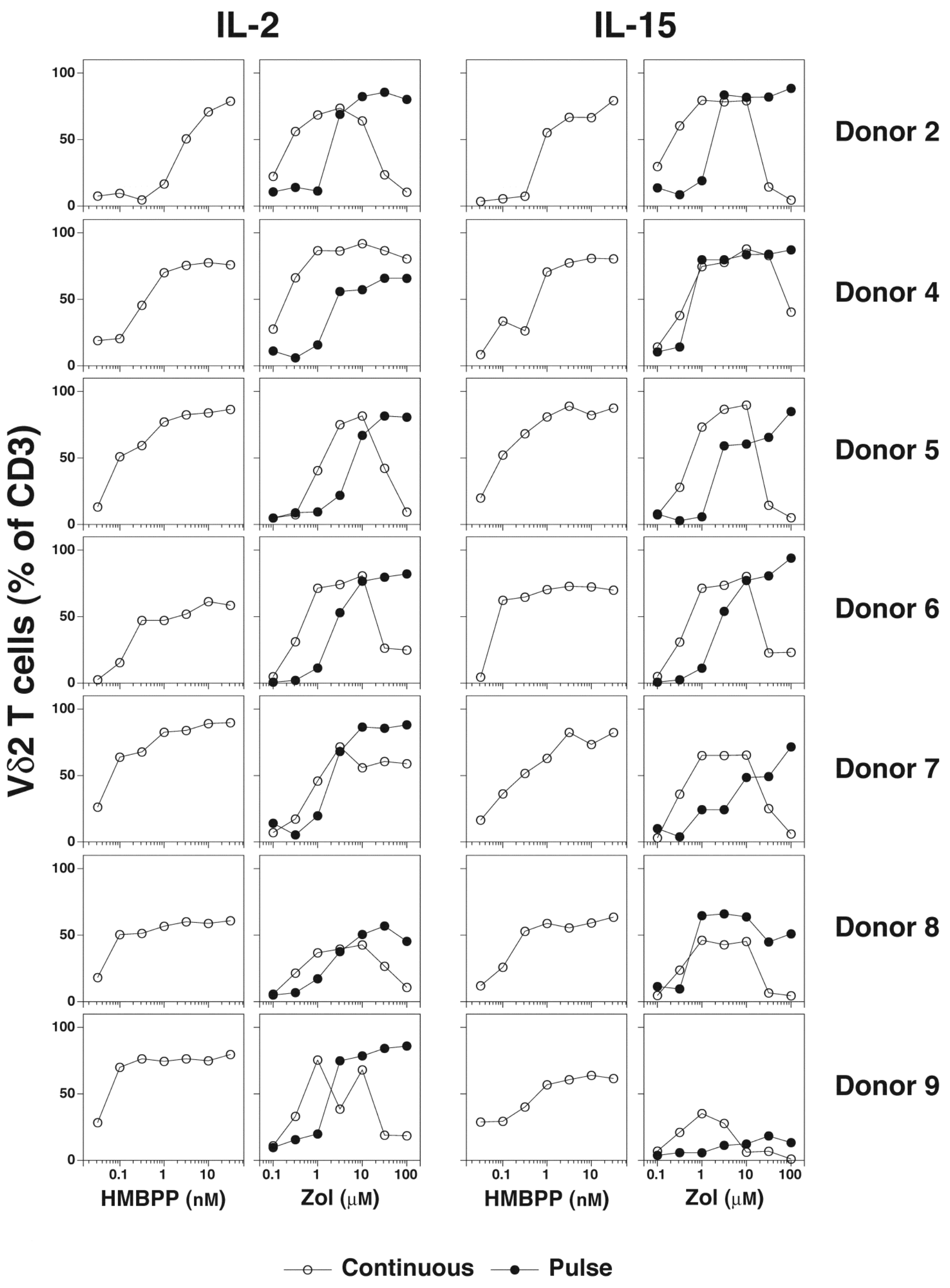


**Figure S4.** Comparison of the expansion of Vγ2Vδ2 T cells in response to varying doses of HMBPP and zoledronate with IL-2 or IL-15. Data are from Fig. 1 and Fig. 5. Human Vγ2Vδ2 T cells were expanded ex vivo from PBMC from each donor by exposure to HMBPP or zoledronate in parallel. Human PBMC were cultured in 96-well plates either continuously with varying starting concentrations of HMBPP or zoledronate (open circles) or pulsed with zoledronate for 4 h (closed circles) followed by washing twice. IL-2 or IL-15 was added to 1000 IU for IL-2 or 100 ng/ml for IL-15 on day 3. Thereafter, media with the respective cytokine was changed every 2-3 d depending on cell growth. On day 14, Vγ2Vδ2 T cell numbers were determined by flow cytometric analysis.

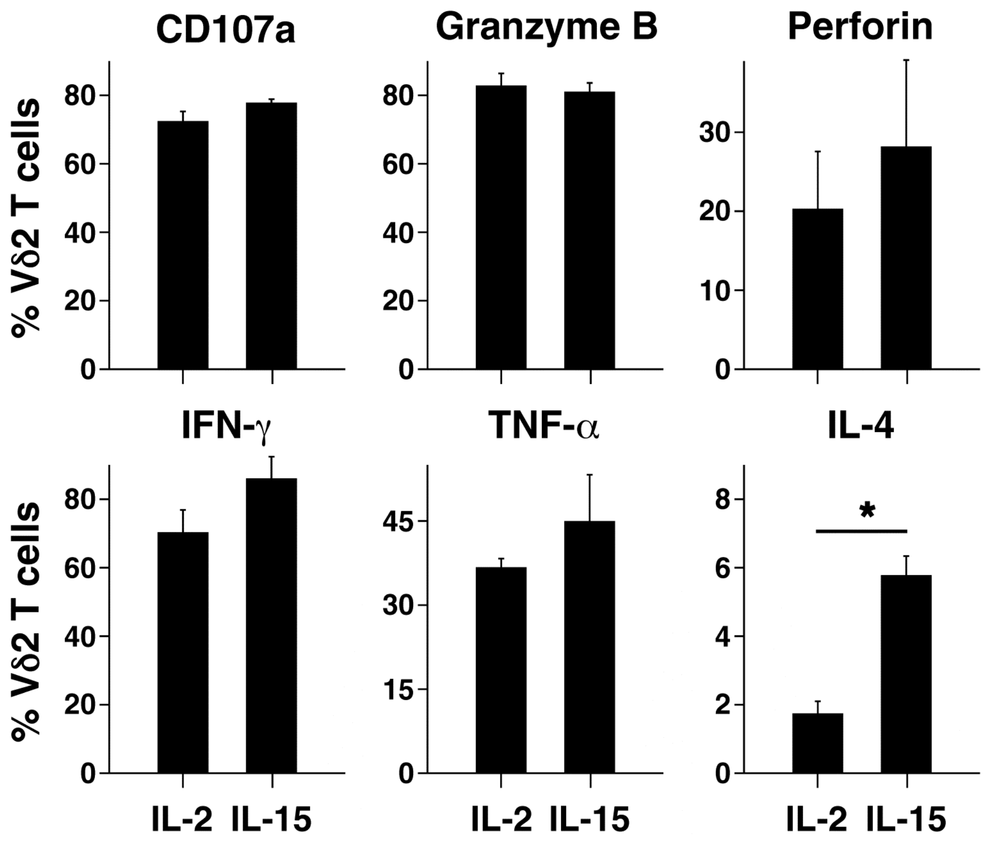


**Figure S5.** Functional capabilities of Vγ2Vδ2 T cells expanded by pulse zoledronate stimulation with IL-15 are similar to those expanded with IL-2. Human PBMCs were pulsed with zoledronate (100 μM) for 4 h and then washed twice before re-culture either with IL-15 (50 ng/ml) or IL-2 (1000 IU/ml) for 14 d. Expanded Vγ2Vδ2 T cells were purified by positive selection or left unpurified. PC-3 cells were treated overnight with pamidronate (200 μM) and then washed. Pamidronate-treated PC-3 cells were incubated with unpurified or purified Vγ2Vδ2 T cells for 4 h in duplicate samples with monensin followed by surface and intracellular mAb staining. Staining was assessed by flow cytometric analysis. Mean ± SD is shown. Representative of two experiments. *$p < 0.05$ using the unpaired *t*-test.

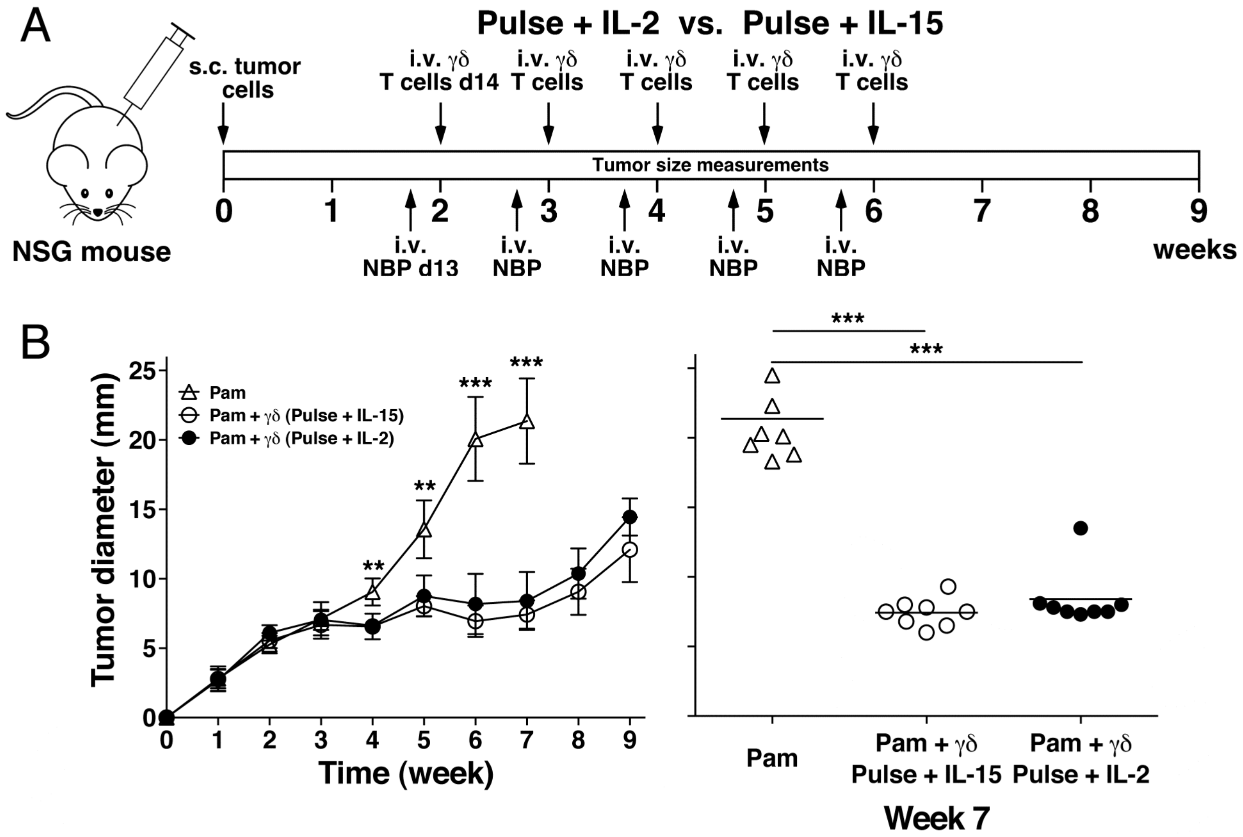


**Figure S6.** Adoptive transfer of Vγ2Vδ2 T cells expanded by pulse zoledronate stimulation with IL-15 in combination with pamidronate controlled PC-3 prostate tumor growth in NSG mice similarly to Vγ2Vδ2 T cells expanded by pulse zoledronate stimulation with IL-2; tumor diameter data for Fig. 7. **a** Schema of treatment protocol used to evaluate the anti-tumor efficacy of Vγ2Vδ2 T cells. Immunodeficient NSG mice were s.c. inoculated with human PC-3 prostate cancer cells on day 0. On day 13, pamidronate (50 µg/kg) was given i.v. On day 14, $1 \times 10^6$ purified Vγ2Vδ2 T cells expanded by pulse zoledronate stimulation with either IL-15 or IL-2 were inoculated i.v. Treatments were repeated weekly until week 6. Longitudinal and transverse diameters of the tumors were measured twice weekly until week 9. **b**, *left panel* Vγ2Vδ2 T cells stimulated by pulse zoledronate exposure with IL-15 showed similar anti-tumor immunity compared with those expanded with IL-2. Mean PC-3 tumor diameter ± SD is shown for 7-8 mice per group treated with either pamidronate alone (open triangles), pamidronate with purified Vγ2Vδ2 T cells derived by pulse zoledronate stimulation with IL-15 (open circles), or pamidronate with purified Vγ2Vδ2 T cells derived by pulse zoledronate stimulation with IL-2 (closed circles). ***$p < 0.001$ compared with mean tumor volume of mice treated with Vγ2Vδ2 T cells derived by pulse zoledronate stimulation with IL-2 using the Mann-Whitney *U* test. *Right panel*, Tumor volume at week 7 of individual mice treated with pamidronate alone (open triangles), pamidronate with Vγ2Vδ2 T cells derived by pulse zoledronate stimulation with IL-15 (open circles), or pamidronate with Vγ2Vδ2 T cells derived by pulse zoledronate stimulation with IL-2 (closed circles). Bars represent mean values. ***$p < 0.001$ using the Mann-Whitney *U* test.
